# Temperature-dependent feedbacks drive the pattern of Antarctic temperature change

**DOI:** 10.1073/pnas.2513383123

**Published:** 2026-05-11

**Authors:** Bradley R. Markle, Eric J. Steig

**Affiliations:** ^a^Department of Geological Sciences, University of Colorado, Boulder, CO 80309; ^b^Institute of Arctic and Alpine Research, University of Colorado, Boulder, CO 80309; ^c^Department of Earth and Space Sciences, University of Washington, Seattle, WA 98195; ^d^Department of Atmospheric Sciences, University of Washington, Seattle, WA 98195

**Keywords:** Antarctica, climate change, paleoclimate, ice cores, climate dynamics

## Abstract

Antarctica is an important component of the Earth’s climate system. Here we investigate temperature change in Antarctica across a range of timescales, from millennial to orbital, over the last $4\times 10^{5}$ y, using a compilation of ice-core water-isotope records. We identify a persistent pattern of change in which the temperature variability of an Antarctic site increases with its mean surface temperature. When the entire continent warms, the warmest parts of Antarctica warm more; when the entire continent cools, the warmest parts cool more. This pattern is inconsistent with the Planck response, the simplest possible null hypothesis for Antarctic temperature change. However, a temperature-dependent feedback explains the fundamental pattern of temperature change. The feedback arises from a nonlinearity of the greenhouse effect, evident only at the cold surface temperatures of the Antarctic. This feedback may be initiated by any mean energetic forcing and thus manifests across all timescales. Local deviations from the expected pattern of temperature change indicate regional forcing such as changes in ice-sheet elevation. We reconstruct the surface elevation of the main ice divide in West Antarctica over the last deglaciation, finding a history that is supported by geological and glaciological evidence and consistent with ice-sheet modeling.

## Significance

Changes in Earth’s climate are caused by changes in incoming and outgoing energy. However, understanding the patterns of temperature response that result from changes in energy balance is not straightforward. Here we investigate the patterns of temperature change within Antarctica. We find a persistent and fundamental pattern of Antarctic temperature change, in which warmer places warm and cool more than colder places. This pattern, caused by a nonlinearity in the greenhouse effect, is observed in essentially all large-scale changes in Antarctic temperature for the last several thousand centuries. By understanding this pattern and the physics that cause it, we better understand the climate of Antarctica as well as changes in the size of the Antarctic ice sheet itself.

The Antarctic is one of the two regions from which the Earth radiates more energy to space than it receives from the sun. It is thus a key component of the climatological heat engine, which moves energy from regions of net gain in the tropics to those of net loss at the poles. The radiative response of the Antarctic to a change in global climate is inherently linked to its surface temperature response. Attention has been devoted to understanding the mechanisms that lead to polar amplification, the tendency of the polar regions to warm and cool more than the tropics, and why it may differ between the Antarctic and the Arctic (e.g., refs. 1 and 2). Less attention has been paid to the pattern of temperature change within the Antarctic and the physical mechanisms that drive it.

**Fig. 1.**
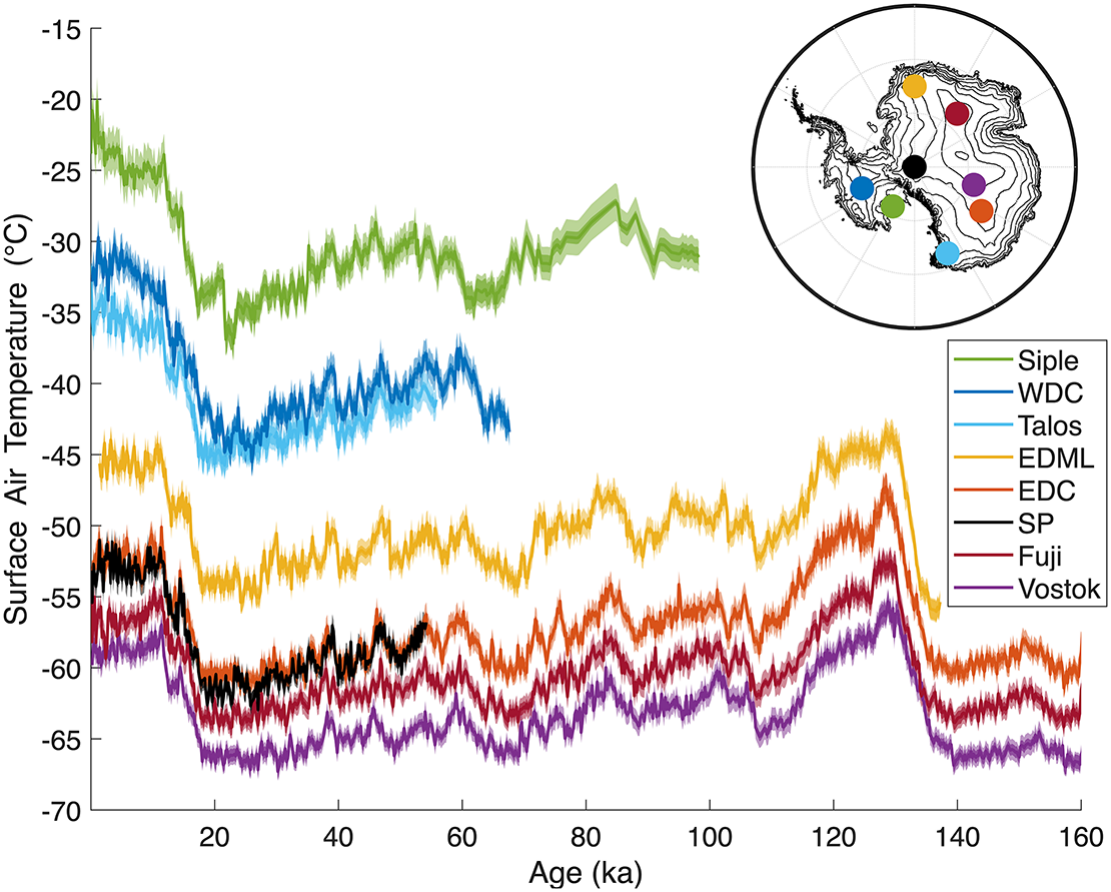
Antarctic temperature reconstructions based on stable-isotope ratios from eight deep ice core records (4). *Inset* shows locations of cores on the Antarctic continent. Raw isotope records shown in *SI Appendix*, Fig. S1.

Temperature proxies from ice cores (Fig. 1), which extend back many millennia, show that the magnitude of temperature change in West Antarctica during the last deglaciation was several degrees greater than in East Antarctica (3, 4). This difference has been attributed to changes in the surface elevation of the West Antarctic ice sheet, with the approximation that without elevation change, the surface temperature response would be uniform across the continent (5–7). However, basic radiative physics suggest that there should be an inherent spatial pattern to Antarctic temperature change.

Temperature changes are caused by changes in energy balance, and even spatially uniform changes in energy balance can drive patterns of temperature response due to spatially variable feedbacks and the redistribution of heat (8–10). The pattern of Antarctic temperature change should be particularly sensitive to feedback processes that are temperature dependent, because Antarctica contains a vast range of surface temperatures. Half the total range of climatological annual mean surface temperatures experienced on Earth occurs in Antarctica.

Here, we evaluate the pattern of Antarctic temperature change, drawing on theoretical expectations and empirical reconstructions of temperature over the past 10^5^ y from stable isotope records from Antarctic ice cores (4) (Fig. 1). We find that there is a characteristic pattern to Antarctic temperature change that persists across a broad range of timescales and is a fundamental consequence of temperature-dependent feedbacks. This result has implications for the interpretation of Antarctic climate variability, as well as our understanding of Antarctic ice-sheet elevation change.

## 1. Theoretical Background

We begin by showing that the basic radiative physics governing Antarctic surface temperature should lead to a spatial pattern in temperature change.

### 1.1. The Planck Response.

The temperature of an idealized surface in radiative equilibrium is determined by the balance between absorbed incoming energy and energy radiated out from the surface, $F_{\mathrm{in}}=F_{\mathrm{out}}$. Following the Stefan–Boltzmann equation, the latter depends nonlinearly on the surface temperature, T:

[1] $$\begin{array}{c} F_{\mathrm{out}}=\epsilon \sigma T^{4}, \end{array}$$

where σ is the Stefan–Boltzmann constant and ϵ is the emissivity (when ϵ=1, Eq. **1** describes the radiation from an idealized blackbody). In response to an energetic forcing, ΔF, the surface warms or cools until the change in outgoing radiation balances the forcing. Differentiating Eq. **1** with respect to temperature shows that the temperature response is a nonlinear function of the surface temperature itself,

[2] $$\begin{array}{c} \frac{\mathrm{dT}}{\mathrm{dF}}=\frac{1}{4\epsilon \sigma T^{3}}. \end{array}$$

Eq. **2** describes the so-called Planck response, the reference sensitivity of the Earth’s climate to a forcing (8, 11), denoted $\lambda_{0}$, upon which more complex feedback processes may also act. For two idealized surfaces of different mean temperature, subjected to an equal change in radiative forcing, the colder surface will require a larger ΔT than a warmer surface to balance the same ΔF.

The temperature sensitivity of the Planck response provides the simplest hypothesis for the pattern of Antarctic temperature change: in response to spatially uniform forcing, we would expect the coldest places to warm or cool more than the warmest places. The Planck response has been invoked previously to explain, in part, the response of Antarctic temperature to orbital-scale Milankovitch forcing (12, 13).

### 1.2. The Greenhouse Effect.

**Fig. 2.**
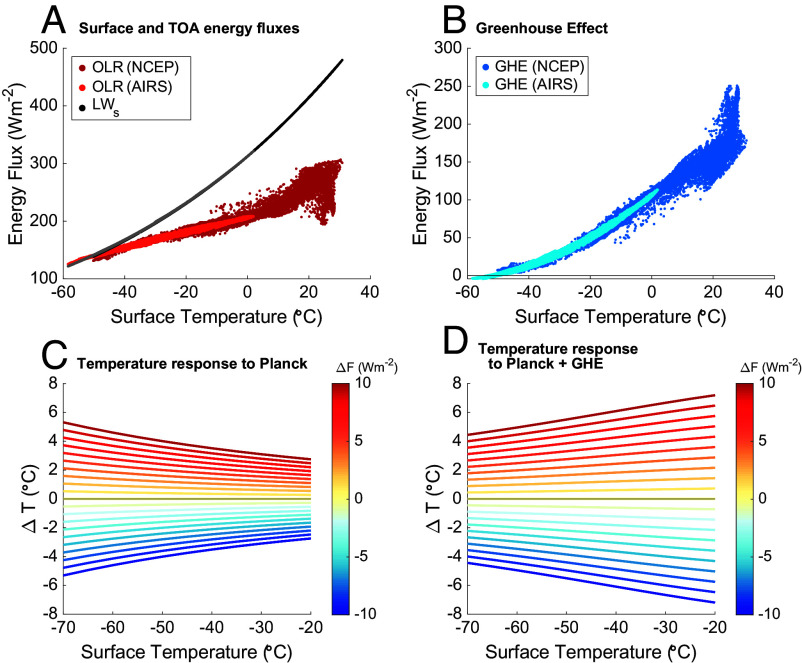
(*A*) Outgoing longwave radiation (OLR) at the top of the atmosphere and upwelling longwave radiation from the surface ($LW_{s}$). OLR data are from monthly NCEP/NCAR reanalysis [red, (17)], and AIRS satellite observations over Antarctica [orange, (18, 19)]. $LW_{s}$ calculated from NCEP/NCAR reanalysis (black) and AIRS data (gray) assuming surface emissivity ϵ=0.99 (both datasets result in essentially identical lines). (*B*) The greenhouse effect calculated as the difference between the OLR and $LW_{s}$ in panel (*A*), as a function of surface temperature. (*C*) Change in surface temperature (ΔT) resulting from the Planck response, as a function of initial temperature, following Eq. **2** for a range of positive and negative forcings (ΔF) indicated by line color. (*D*) As in (*C*), but including the nonlinear greenhouse-effect feedback.

The hypothesis given by the Planck response neglects the role of the atmosphere in local energy balance. Fig. 2*A* shows the longwave radiation emitted from the surface ($LW_{s}=\epsilon \sigma T^{4}$) and observations of the outgoing longwave radiation (OLR) at the top of the atmosphere (TOA), as a function of surface temperature. Their difference is the greenhouse effect ($GHE=LW_{s}-OLR$), which quantifies the additional energy the surface gains owing to the presence of the atmosphere, which absorbs and re-emits longwave radiation (Fig. 2*B*).

The dominant greenhouse gas in the atmosphere is water vapor, the concentration of which is a nonlinear function of air temperature, the Clausius–Clapeyron relationship (14). The radiative effects of water vapor are also nonlinear, though these largely compensate the nonlinearity in Clausius–Clapeyron (15), leading to a nearly linear relationship between surface temperature and the greenhouse effect over most of the Earth, outside the tropics. However, the temperature dependence of the GHE becomes significantly nonlinear at temperatures below about −20 ^°^C, as atmospheric water vapor diminishes. At very cold surface temperatures, typical only of Antarctica, the GHE becomes vanishingly small and Earth’s longwave radiation to space approaches that of a blackbody (Fig. 2 *A* and *B*). The longwave emissivity of snow is very high, typically in the range of 0.98 to 0.99 (16).

The nonlinearity in the greenhouse effect should impact the temperature response of Antarctic surfaces and result in a different spatial pattern than predicted by the Planck response alone. Consider some positive change in energetic forcing, ΔF, that leads to an initial positive temperature response ΔT. That increase in temperature will increase the water vapor content and alter the temperature profile of the atmospheric column to increase the local greenhouse effect, which amplifies the initial temperature response. If the GHE were strictly linear with surface temperature, the gain would be the same for any initial surface temperature, equally amplifying any initial ΔT. However, because the GHE is nonlinear over the range of Antarctic surface temperatures ($\frac{d}{\mathrm{dT}}GHE$ increasing with T), the warmest Antarctic sites will experience the largest change in GHE per increase in surface temperature and therefore the greatest amplification of initial warming.

This describes a temperature-dependent feedback. The greenhouse effect results from a variety of fundamental processes in the atmospheric column, including mechanisms that are usually partitioned as separate feedbacks. The magnitude of the temperature dependence of $\frac{d}{\mathrm{dT}}GHE$, diagnosed from observations or reanalysis [Fig. 2 and *SI Appendix*, Fig. S2, (17–19)], is accurately predicted by radiative-convective equilibrium models [*SI Appendix*, Fig. S6, (20–23)]. It is also consistent with the relationship between surface temperature and the sum of the lapse-rate, water-vapor, and shortwave feedbacks over the Antarctic continent, as diagnosed from a comprehensive atmospheric climate model [CESM-CAM5, (24), *SI Appendix*, Fig. S7].

Treating the temperature-dependent slope of the greenhouse effect, $\frac{d}{\mathrm{dT}}GHE$, as a feedback whose magnitude is a function of the initial surface temperature, we calculate an expectation for the temperature response to any given forcing, for a range of initial surface temperatures. Following Roe (8), the total temperature change resulting from a feedback, c, and an initial forcing, ΔF, is

[3] $$\begin{array}{c} \Delta T=\frac{\lambda_{0}\Delta F}{1-c\left(T\right)\lambda_{0}}, \end{array}$$

where $\lambda_{0}$ is the reference Planck response (Eq. **2**). Here, c(T) is the additional greenhouse effect gained as a function of the temperature response ($\frac{d}{\mathrm{dT}}GHE$), found from the derivative of a second-order polynomial fit of GHE to surface temperature in the AIRS observational dataset [*SI Appendix*, Fig. S4, (18, 19)]. We ignore the negligible second-order terms in the response (8, 25). Fig. 2 *C* and *D* illustrate the temperature response to a set of positive and negative forcings, given a range of initial Antarctic surface temperatures, for the Planck response alone (panel *C*), and taking into account the nonlinearity of the GHE (panel *D*) acting as a feedback on top of the Planck response. As the comparison of Fig. 2 *C* and *D* shows, the feedback associated with the GHE is large enough to overwhelm the temperature-dependent tendency of the Planck response, resulting in larger temperature change at warmer sites rather than at colder sites.

The temperature dependence of the GHE feedback provides a simple, testable hypothesis for Antarctica. In response to equal forcing, we should expect the warmest places to warm or cool more than the coldest places, and to do so at predictable relative magnitudes. Importantly, this response to the GHE feedback should be independent of the source of the forcing (e.g., whether it is from top-of-the atmosphere radiative forcing, atmospheric heat convergence, or other combinations of mechanisms). It should also be independent of timescale, as it arises from fast atmospheric feedbacks. The alternative null hypotheses are either that there is no particular spatial pattern, or that the pattern more closely follows the Planck response. We now turn to evaluating these competing predictions by comparison with the empirical observations from ice cores.

## 2. Results

### 2.1. Observations.

To examine spatial variability in temperature across the Antarctic continent, we rely on ice-core records of the concentrations of oxygen and hydrogen isotopologues in water (“water-isotope ratios”). Water and its phase changes play a fundamental role in almost all aspects of the energetics of the climate system (26, 27). The transport of water in the atmosphere leads to progressive distillation and integrated fractionation of water-isotope ratios in vapor and precipitation. These processes relate variability in water-isotope ratios in precipitation to variability in temperature at high-latitude sites (27–30).

To reconstruct Antarctic surface air temperatures and their uncertainties, we use the technique outlined by Markle and Steig (4), which uses both the oxygen and hydrogen isotope ratios to account for the influence of changes in conditions at the moisture evaporation source as well nonlinearities in water-isotope distillation (Fig. 1). We use eight long ice-core water-isotope records from Antarctica (*SI Appendix*, Fig. S1). All cores have been placed on the most recent published age scales, most of which have been synchronized (31, 32). The use of water isotopes to determine quantitative temperature histories in Antarctica is supported both by more complex numerical modeling than we use here, and by independent empirical measurements of borehole temperatures, inverted for the effects of ice advection and heat diffusion (3, 4, 33).

### 2.2. The Last Deglaciation.

**Fig. 3.**
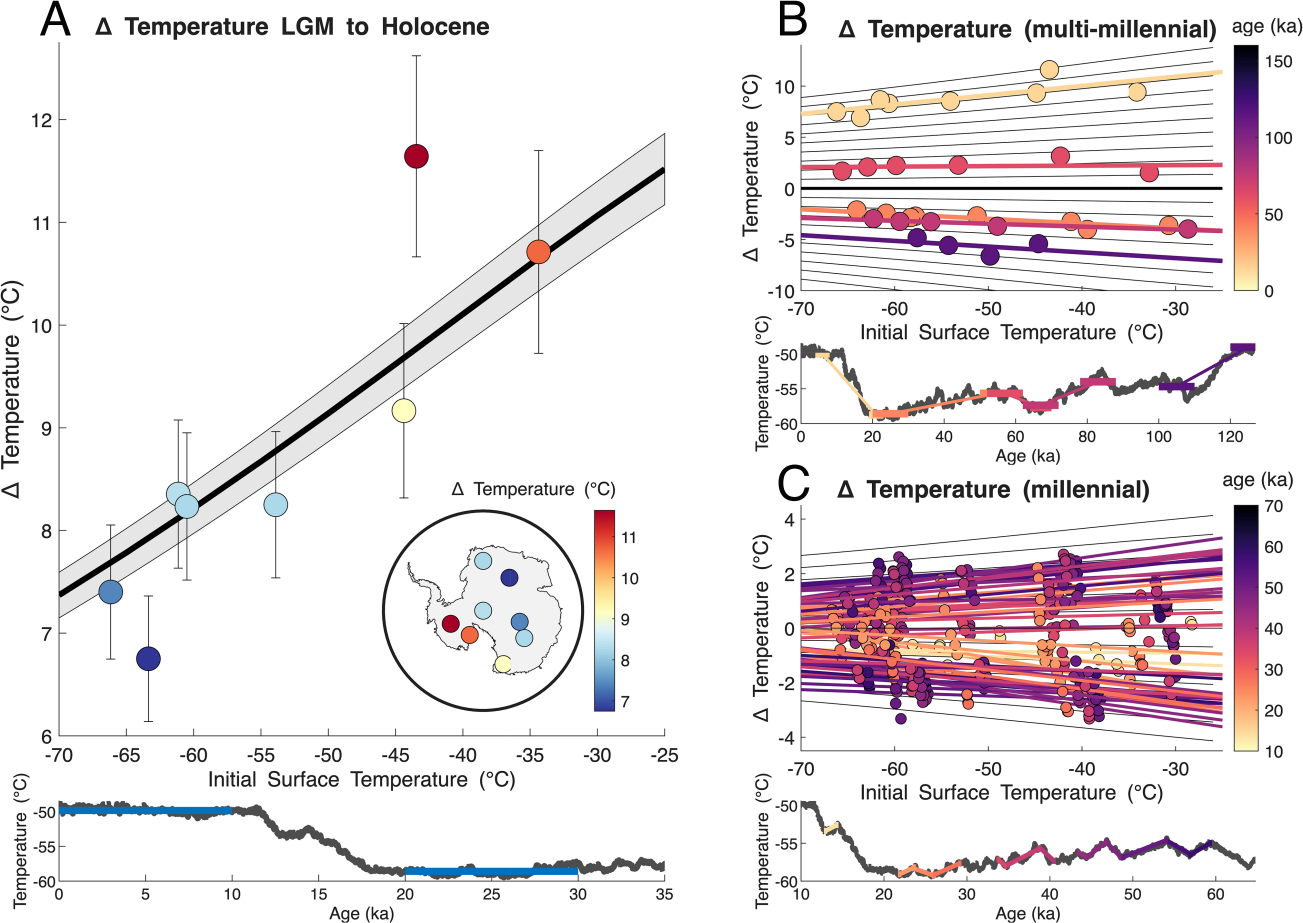
Change in temperature as a function of initial temperature, from ice-core temperature reconstructions, for different timescales. (*A*) Pattern of warming between the LGM and Holocene across Antarctica as a function of LGM surface temperature. Circles show the temperature change for each ice-core site, with 2 SD uncertainty (4). *Inset* shows the site locations, colored by magnitude of warming. The diagonal black line shows the pattern of temperature response expected from a single forcing amplified by GHE, as in Fig. 2, given the mean temperature change of all sites. Shading shows the spread from ±0.5 Wm^−2^ on the forcing. Lower subpanel shows mean Antarctic temperature since 35 ka; blue bars indicate the intervals between which the temperature change was calculated. (*B*) As in panel (*A*) but for several distinct intervals of continent-wide mean temperature change occurring over >10 ka timescales. Circles show magnitude of warming at each site, with best-fit linear regressions, colored by mean age of the interval. Black lines show the predicted GHE response to a range of forcing as in Fig. 2*D*. *Lower* subpanel indicates the intervals between which the temperature changes were calculated, colored by mean age. (*C*) As in panel (*B*) but for millennial-scale AIM events. Because of the shorter nature of the events and potential influence of noise, we include several different block lengths of the time periods to be averaged and then differenced (200, 300, 500, and 1,000 y blocks). Black lines show the predicted GHE response to a range of forcing as in Fig. 2*D*. *Lower* subpanel indicates the intervals over which the temperature changes were calculated, colored by mean age.

We first examine the large temperature change that occurred between the last glacial maximum (LGM, about 20 ka) and the Holocene (starting at 11.7 ka). This deglaciation was driven by changes in top-of-atmosphere insolation due to changes in Earth’s orbit, amplified by climate feedbacks including changes in albedo (associated with the disintegration of large Northern Hemisphere ice sheets and reductions in sea ice and land snow cover), increases in greenhouse gases, and other changes in energy balance. Our reconstructions show a coherent change in temperature across Antarctica in response to these forcings and feedbacks. The mean change in surface temperature for the eight ice-core records in Fig. 1 is 8.8 ^°^C, consistent with earlier work (e.g., ref. 34), calculated as the difference between the average temperature in the interval 10 ka to 0 ka and the interval 30 to 20 ka. The magnitude of the warming since the LGM varied across the continent, ranging from 6.8 ^°^C at Dome Fuji to 11.6 ^°^C degrees at WAIS Divide (Fig. 3*A*) (4). The central LGM-to-Holocene temperature-change estimate at WAIS Divide from borehole temperature inversion (3) is 11.8 ± 1.3 ^°^C, in excellent agreement with our results (11.2 ± 1.0 ^°^C, when calculated over the same time interval as ref. 3). Other estimates of the magnitude of this change, which rely on firn modeling (7, 35), yield somewhat smaller amplitude changes at some sites, but a similar general pattern.

We find that the initial (or the time-mean) absolute temperature at each ice-core site is a strong predictor of the magnitude of temperature change between the LGM and Holocene (Fig. 3*A*). The warmer the site, the greater the warming since the LGM. The site elevation is also a strong predictor of total warming, since surface temperature and elevation are inherently related. Temperature is a better predictor than other variables that might be thought important, such as the latitude, longitude, or distance of the site from the coast. Critically, the temperature-dependent pattern is not simply a result of differences between West and East Antarctica. Indeed, initial surface temperature is a strong predictor of the temperature response even if we consider only records from East Antarctica. This key result—that the magnitude of warming at a site during the deglaciation is function of its absolute temperature—is also insensitive to other specific choices in the analysis. For example, using narrower intervals to define the last deglaciation (e.g., from 4–8 ka to 19–23 ka) leads to the same pattern (gold circles and line Fig. 3*B*). This pattern is also apparent in the raw water-isotope records, in which the magnitude of change in $\delta^{18}$O is a strong function of the initial $\delta^{18}$O (*SI Appendix*, Fig. S2).

### 2.3. Timescale Independence.

We examine multiple distinct periods of continent-wide, 10 ka-or-longer intervals of large-scale surface temperature change in Fig. 3*B*. We find that the initial surface temperature is consistently a strong predictor of the temperature change at each ice-core site when there is a coherent change in mean Antarctic temperature. For example, during the interval of continent-wide cooling into the LGM, from about 60 to 20 ka, the temperature cools at all sites, and the sites with the warmest initial temperature cool more than sites with colder initial temperatures (Fig. 3*B*, orange line and circles). For each interval examined, we plot the temperature change at each site in circles as well as a best-fit line between the magnitude of temperature change and the initial surface temperature (Fig. 3*B*). We find that when the whole continent warms, the warmest sites warm more, and when the entire continent cools, the warmest sites cool more. This characteristic relationship holds even in the small subset of East Antarctic records that extend further back in time, including the penultimate deglaciation (*SI Appendix*, Fig. S3).

This relationship is persistent at shorter timescales. In particular, the same dependence of the temperature response to initial temperature is observed for the millennial-scale variations known as the Antarctic isotope maximum (AIM) events (Fig. 3*C*). The AIM events are the Antarctic counterparts to the abrupt climate changes in the Northern Hemisphere known as Dansgaard–Oeschger events (36–38). These events are driven by a different set of processes (e.g., ref. 39) than the Milankovitch orbital forcing that drives longer-term climate changes such as the LGM-to-Holocene transition, providing clear evidence that the pattern of the Antarctic temperature response is independent of the nature of the forcing.

### 2.4. Hypothesis Testing.

Our findings are clearly not consistent with either the hypothesis that Antarctica warms and cools uniformly, nor that Antarctica simply follows the Planck response (compare Fig. 2*C* to Fig. 3). However, our findings are very much in line with expectations from the nonlinearity of the greenhouse effect. The diagonal black line in Fig. 3*A* shows the temperature response to a single mean forcing (16.6 Wm^−2^), locally amplified by the GHE feedback following Eq. **3**; likewise the black lines in Fig. 3 *B* and *C* show the pattern of temperature change expected in response to a range of forcing amplified by the GHE feedback as in Fig. 2*D*.

To rigorously test the GHE explanation for the temperature response pattern, we systematically investigate all intervals of mean temperature change across the continent. We calculate the average temperatures, $T_{1}$ and $T_{2}$, of two blocks of temperature data of length x years separated by a time interval of y years, as well as the change in temperature between those blocks, ΔT, at every site, and the mean change in continent-wide temperature across all sites, $\bar{\Delta T}$. We vary the block size x from 1 to 10 ka in steps of dx= 1 ka, and the separation y from 2 to 40 ka. We choose progressive steps of dy= 1 ka for y≤10 ka, dy=2 ka for 12≤y≤22 ka, and dy=5 ka for 25≤y≤40 ka. We slide these blocks across the time interval 0 to 70 ka, in steps of dt= 1 ka. We thus examine a total of $1.35\times 10^{4}$ intervals of continent-wide temperature changes across a vast range of timescales, spanning the Holocene and last glacial period.

**Fig. 4.**
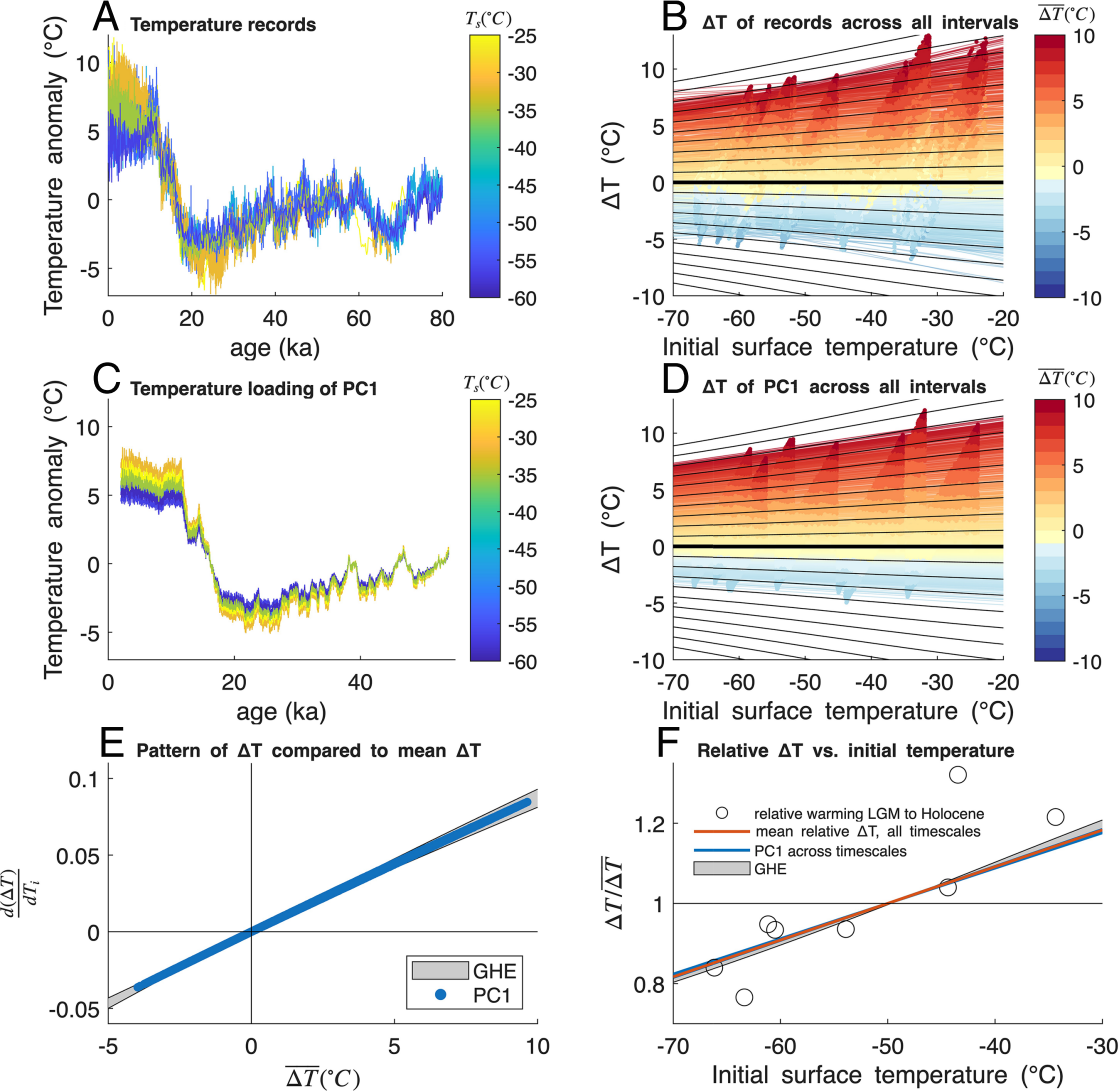
Systematic analysis of the pattern of temperature change. (*A*) Temperature anomaly from ice-core reconstructions, colored by mean Holocene temperature, $T_{s}$. (*B*) Temperature change, ΔT, as a function of initial surface temperature (dots) with best-fit lines, colored by the mean temperature change across all sites ($\bar{\Delta T}$) over each time interval. Dots are shown for all $1.35\times 10^{4}$ distinct time intervals, but only every 5th best fit line is shown, for visual clarity (*SI Appendix*, Fig. S9 shows all results). Black diagonal lines show expectation from the greenhouse effect as in Fig. 2. (*C*) Temperature loading of PC1 for all sites, colored by mean Holocene surface temperature, $T_{s}$. (*D*) As in (*B*), but for PC1. (*E*) Slope of the temperature pattern ($\frac{d(\Delta T)}{dT_{i}}$) as a function of Antarctic mean temperature change ($\bar{\Delta T}$). Blue dots show the results for PC1, equivalent to the derivative of all colored lines in panel (*D*). Gray shading is the prediction from the greenhouse effect, with the range reflecting the uncertainty in the polynomial fits to the AIRS observations (see *SI Appendix*, Fig. S4 and Section 5, *Materials and Methods* for details). (*F*) Relative temperature change as a function of initial temperature. Circles show the relative temperature change across all ice core sites for the LGM to Holocene transition (Fig. 3*A*). The red line shows the mean relative temperature change of the best-fit lines across all timescales from panel (*B*), for intervals of significant continent-wide temperature change. The blue line shows the results for PC1 [all lines in panel (*D*)], and gray shading shows the prediction from the greenhouse-effect feedback.

For each interval, we fit a line via linear regression to the magnitude of warming at each site, ΔT, as a function of the initial temperature at each site, $T_{i}$. The slopes of these best-fit lines, $\frac{d(\Delta T)}{dT_{i}}$, quantify the pattern of temperature change among sites. The results are shown in Fig. 4*B* and are robust to choices in x, y, dx, dy, and dt. The overriding pattern across all intervals of temperature change is clearly evident in Fig. 4*B*, and consistent with the pattern identified from intervals selected a priori in Fig. 3: the warmer parts of Antarctica experience larger temperature changes than the colder parts, across all timescales, whether the whole continent warms or cools on average. This can be seen in the best-fit lines for each interval fanning-out to the right in Fig. 4*B*, just as in the temperature response pattern predicted by the GHE feedback (black lines in Fig. 4*B*). This pattern is robust to the temperature reconstruction technique and also evident in the raw water isotope records (*SI Appendix*, Fig. S10). In Section 5, *Materials and Methods* and *SI Appendix*, Fig. S11 we show that across all timescales, the sign of the pattern across sites ($\frac{d(\Delta T)}{dT_{i}}$) is extremely well predicted by the mean temperature change, which holds if we restrict our analysis only to records from the East Antarctic ice sheet.

This fundamental pattern of temperature change can be quantified further by principal component and empirical orthogonal function (PC/EOF) analysis, which we conduct on the temperature reconstructions back to 54 ka (the maximum overlap of all records). The leading mode of variability in all records (PC1) explains 95.8% of the total variance and shows coherent variability across the continent (Fig. 4*C* and *SI Appendix*, Fig. S8), very similar to the simple mean of all records. PC1 is the only mode with strong loading of the same sign across all sites. The spatial loading, EOF1, of this mode is a strong function of mean temperature, with warmer sites showing greater variability (*SI Appendix*, Fig. S8), and as we show below, is extremely well predicted by the GHE feedback. The leading mode of variability identified in PC1/EOF1, together with the analysis of reconstructions across millennial to multimillennial timescales, shows that the pattern of temperature change between sites is in phase with the mean change across sites.

The remaining principal components explain little total variance, and reflect the unshared variability of individual sites (*SI Appendix*, Fig. S8). For example, the second mode, PC2, contains the unique abrupt variability at Siple Dome (40), while PC3 shows unique variability at WDC (13, 38), explored in detail below.

We conduct the same sliding-block analysis as above on the leading mode of temperature variability at all sites (PC1/EOF1, Fig. 4*C*), calculating ΔT, $\bar{\Delta T}$, and $\frac{d(\Delta T)}{dT_{i}}$, for $9.7\times 10^{3}$ intervals of continent-wide temperature change. This allows us to better evaluate the ability of the GHE feedback to explain the leading pattern of shared temperature variability among sites. The result (Fig. 4*D*) is both consistent with the pattern in the raw reconstructions (Fig. 4*B*) and extremely well predicted by the greenhouse effect (black lines in Fig. 4*D* show the expectation from the GHE). The slopes of best fit ($\frac{d(\Delta T)}{dT_{i}}$) during periods of continent-wide mean warming ($\bar{\Delta T}>0$) and cooling ($\bar{\Delta T}<0$) are perfectly separated (*SI Appendix*, Fig. S12): all intervals of mean warming have a positive $\frac{d(\Delta T)}{dT_{i}}$ slope, while all intervals of mean cooling have a negative slope. These results are predicted by the nonlinearity of the GHE and cannot be reconciled with the Planck effect acting alone, nor with uniform change across the continent.

As is evident in Figs. 3 and 4 *B* and *D*, the strength of the pattern of temperature change is a function of the Antarctic-mean temperature change: the larger the mean change in temperature ($\bar{\Delta T}$), the greater the difference in ΔT between the warmest and coldest sites, and the steeper the slope $\frac{d(\Delta T)}{dT_{i}}$, whether it is positive or negative. This finding is also consistent with the GHE feedback, which not only predicts that the sign of the pattern of temperature change depends on whether the continent warms or cools on average, but that the magnitude of the pattern depends on the magnitude of the mean warming or cooling. In Fig. 4*E* we compare the slope of the pattern across sites, $\frac{d(\Delta T)}{dT_{i}}$, to the mean temperature change of all sites, $\bar{\Delta T}$, for each of the $9.7\times 10^{3}$ intervals of change in PC1 examined above (blue dots). The observed relationship between $\frac{d(\Delta T)}{dT_{i}}$ and $\bar{\Delta T}$ in PC1 is predicted by the greenhouse effect (shown in gray shading, Fig. 4*C*) with very high precision. The bounds of the GHE prediction in Fig. 4*C* derive from the 95% confidence bounds of the temperature dependence of GHE estimated from both monthly and annual averaged AIRS data and calculations using Eq. **3**. We calculate the coefficient of determination for the GHE feedback prediction of the relationship between $\frac{d(\Delta T)}{dT_{i}}$ and $\bar{\Delta T}$ in PC1, using the upper and lower bounds of the GHE prediction as well as the mean, finding $R^{2}>0.999$ in all cases. This indicates that the nonlinearity in the greenhouse effect entirely accounts for the leading pattern of variability among sites.

Finally, we examine the relative temperature change between sites by dividing ΔT at each site by $\bar{\Delta T}$, the mean temperature change across all sites. Fig. 4*F* shows the relative temperature change across all sites for the LGM to Holocene transition as a function of their initial temperature (black circles). We show the average relative temperature change ($\text{Δ}T/\bar{\text{Δ}T}$, red line Fig. 4*F*) of all the lines of best fit of the sliding block analysis (Fig. 4*B*), for all intervals of significant continent-wide temperature change (when $|\bar{\Delta T}|>0$ ^°^C, P<0.05). The relative change of all $9.7\times 10^{3}$ lines of best fit from the systematic analysis of PC1 (Fig. 4*D*) are shown in light blue lines in Fig. 4*F*. These patterns are all in close agreement, and together show that, whether warming or cooling, the warmest parts of Antarctica are more than 20% more variable than the Antarctic mean, while the coldest sites are about 20% less variable. This magnitude of relative variability in Antarctic temperature is exactly predicted by the greenhouse effect, shown in gray shading in Fig. 4*F* [$R^{2}>0.99$ for the mean GHE prediction to the mean relative temperature change in both the reconstructions (red line) and PC1 (blue lines)].

We conduct the sliding-block and principal component analysis on the EDC, Fuji, and Vostok records, which span the last 400 ka (*SI Appendix*, Fig. S13). This analysis confirms the essential results above: |ΔT| increases with initial surface temperature, across a range of timescales, throughout the last four glacial cycles.

## 3. Discussion

### 3.1. Spatial-Temporal Scales.

Our results show that, across a range of timescales, the pattern of Antarctic temperature change is a function of the absolute surface temperature, and that the nonlinearity of the GHE feedback accounts for the overwhelming majority of the differences among Antarctic ice-core temperature records. The pattern of temperature response does not correspond with any known pattern of external forcing. For example, while orbital forcing is a function of latitude, the temperature response is not. Furthermore, the orbital forcing that dominates the longest timescales is distinct from the variety of forcing mechanisms that lead to variability on shorter (e.g., millennial) timescales. Yet the same temperature response pattern emerges across all timescales. This is expected if a fast-acting feedback process, directly related to surface temperature, is locally amplifying any forced changes.

Of course, not all of the spatial variability in Antarctic temperature change can be attributed solely to the GHE feedback. For example, while the GHE feedback clearly drives the pattern on millennial timescales, there is also spread in the response. Millennial-scale temperature variations in Antarctica are related to, among other things, variations in meridional atmosphere and ocean heat transport that may be zonally asymmetric (38, 39), which may lead to regional variation in forcing. At the shortest timescales, the observed warming over West Antarctica and the Antarctic Peninsula in recent decades (41–43) is related to changes in atmospheric circulation, increases in anthropogenic greenhouse gases, loss of stratospheric ozone, and tropical-extratropical teleconnections (e.g., refs. 44 and 45). While the GHE feedback may amplify these combined forcings, contributing to a similar temperature response pattern to the one we identify at longer timescales (42), spatial inhomogeneity in the forcing is likely large compared to the mean forcing.

These observations can be understood by considering the feedback factor, $f=\lambda_{0}c$, which quantifies the fraction of the initial forcing that is fed back into the system. At the coldest Antarctic temperatures, the feedback factor related to the nonlinearity of GHE is near zero, or slightly negative, meaning that the temperature response is close to the reference Planck response. However, at the warmest Antarctic temperatures, the GHE feedback factor is strongly positive, near to 0.6 (*SI Appendix*, Fig. S5). The gain of the system, which quantifies the ratio between total temperature change with and without the feedback, scales with $\frac{1}{\left(1-f\right)}$ (8). Thus, by the time the warmest parts of the continent have reached a new equilibrium, the feedback will have more than doubled the initial energy imbalance to which the system must respond, leading to a local temperature change up to two and half times larger than it would be without the GHE feedback. Over intervals of large mean change in Antarctic temperature and over longer timescales, the mean forcing must be large compared to inhomogeneity in the forcing. In such cases, the amplification due to the GHE feedback will dominate the spatial pattern of temperature change. The imprint of the GHE feedback will be less pronounced if the gain due to the feedback is less than the ratio of the spatial variability in the forcing to the mean forcing, as is likely at very short timescales or over intervals of small changes in mean temperature.

### 3.2. Changes in Ice Sheet Height.

We now consider the implications of our results for estimates of past ice-sheet elevation. To the extent that local accumulation changes follow the Clausius–Clapeyron relationship (e.g., ref. 46), they could drive changes in ice-sheet elevation in phase with changes in mean climate, the magnitude of which would depend on local temperature. Because of the surface lapse rate, such changes in elevation could result in different surface temperature changes between sites. However, because of the nonlinearity in the Clausius–Clapeyron relationship, these accumulation-driven changes in ice sheet height should tend to damp apparent temperature changes at warmer sites compared to colder sites, resulting in a pattern of relative surface temperature change opposite to the one we identify. Further, dynamic changes in the ice sheet and those driven by ice loss at the margin should lag the climate forcing by thousands of years owing to the ice sheet’s characteristic response times, which vary between the East and West Antarctic Ice Sheets (47). Yet the temperature response pattern we identify consistently emerges across a range of timescales, both within EAIS and between EAIS and WAIS, and is not lagged in time compared to the continental average. It is thus unlikely that the pattern of Antarctic temperature response identified here can be attributed primarily to changes in ice-sheet elevation.

Of course, the ice sheets do change with climate and the GHE feedback does not explain all the differences in temperature variability among sites. Indeed, we identify unshared patterns of temperature variability at all sites (*SI Appendix*, Fig. S8). A prominent point of deviation from the shared Antarctic pattern is the magnitude of the glacial-interglacial warming observed in the WAIS Divide ice core (WDC) record, which not only warmed more than East Antarctic sites but warmed more than predicted by the GHE feedback (Fig. 3*A*).

If we assume that the deviation of the temperature change at WDC from the expected pattern (owing to the GHE feedback) can be attributed to changes in ice sheet thickness, we can estimate the magnitude of the associated surface elevation changes through time. To do this, we subtract the first principal component of all temperature reconstructions (PC1 weighted for the WDC site) from the total WDC temperature reconstruction; this yields residual temperature variability at WDC not explained by the GHE feedback. We obtain essentially identical results if we instead use the temperature variability associated with PC3, the leading mode of unshared variability unique to WDC. Assuming the anomalous temperature variability at WAIS Divide entirely arises from local elevation changes, we calculate the latter assuming a dry adiabatic lapse rate (γ=9.8^°^ C/km), which is reasonable for the surface lapse rate in this region of Antarctica (48, 49).

**Fig. 5.**
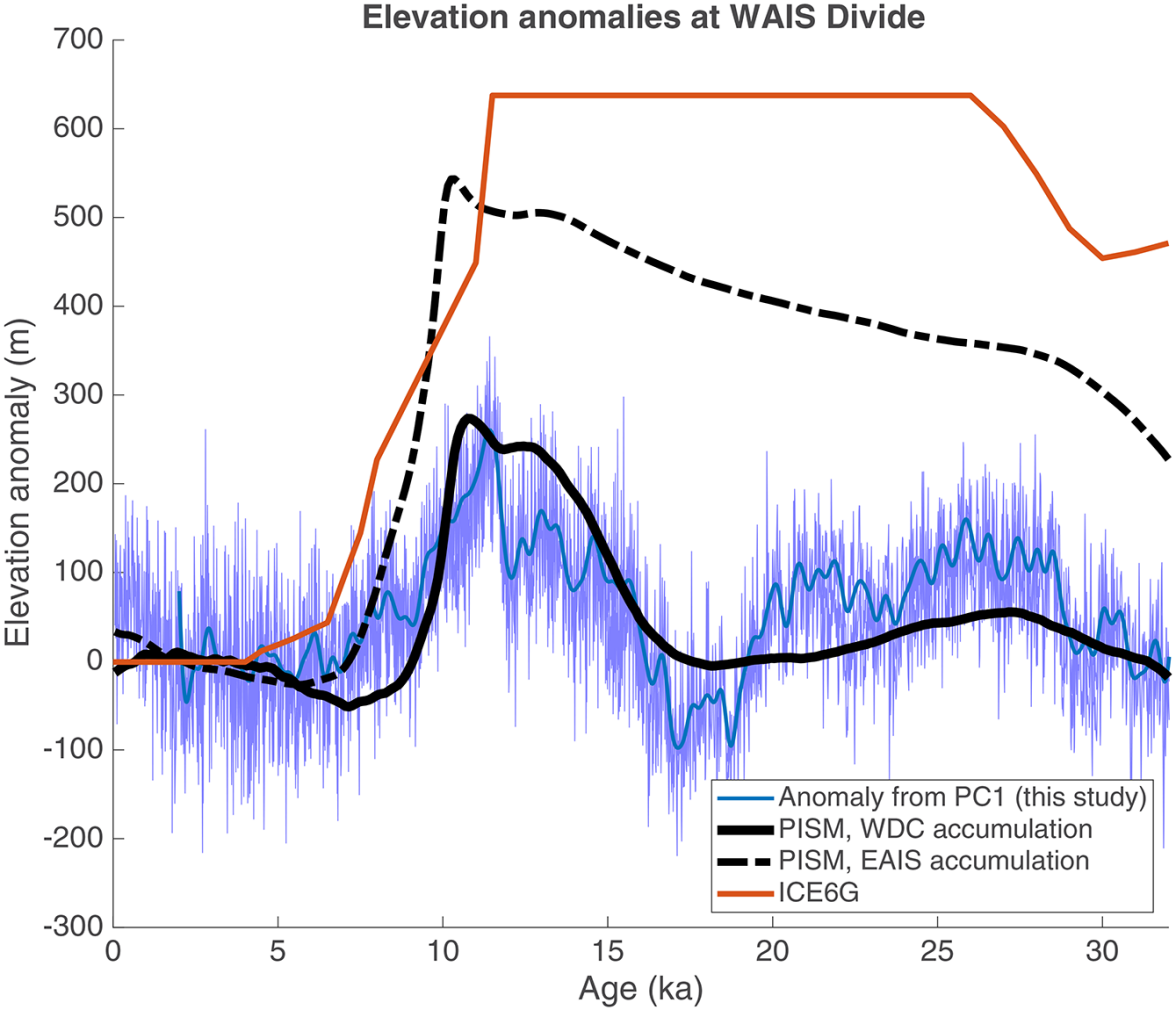
Reconstructions of elevation changes at WAIS Divide, West Antarctica. Dark blue shows the variability in the WDC ice-core record in excess of PC1, converted to a relative surface elevation change assuming a fixed lapse rate ($\gamma =9.8^{{}^{\circ}C}\mathrm{km}$). The bold blue line shows the same data with frequencies higher than 1500 y removed by fourth-order Butterworth filter. An alternate calculation using PC3 is shown in *SI Appendix*, Fig. S14. The black dashed line shows an ice-sheet model (PISM) estimate of elevation change at the WDC site (52, 53), using estimates of accumulation changes through time based on East Antarctic ice-core records. The solid black line shows a reconstruction using the same PISM configuration but using an estimate of the accumulation change from the WDC ice-core record (52, 53). The red line shows the elevations changes from the ICE6g reconstruction at WAIS Divide (56).

The results, shown in Fig. 5, indicate an increase in ice-sheet elevation at WAIS Divide between the LGM and the start of the Holocene of about 250 m, followed by thinning through the Holocene. The results are robust to reasonable choices in the surface lapse rate, including superadiabatic lapse rates seen on the high East Antarctic plateau (48) (*SI Appendix*, Fig. S14). This reconstructed surface elevation change is consistent, in both timing and magnitude, with geological evidence from exposed bedrock which shows that the ice-surface elevation within a few hundred km of the main West Antarctic ice-sheet divide, where WDC was drilled, was up to about 200 m thicker than present at about 10 ka (47, 50, 51) and subsequently thinned. The temporal pattern of change is also physically plausible given the accumulation history from WDC, in which accumulation more than doubled between 20 ka and 12 ka (13), which should have thickened the ice sheet initially, before dynamic flow or other changes at the ocean boundary led to subsequent thinning in the Holocene.

This result is also consistent with the borehole temperature measurements from WAIS Divide, which were used by Cuffey et al. (3) to invert for surface temperature using a combination of ice-flow modeling and gas and water-isotope constraints. Cuffey et al. (3) consider an upper limit on ice thickness change, noting that thinning is associated with increased vertical ice velocity, which would shift the resulting borehole temperature profile; LGM thickness changes in excess of 300 m produce temperatures incompatible with the borehole observations. Moreover, isostatic adjustment means that any change in ice thickness will always yield a smaller change in surface elevation. Thus, the borehole temperature measurements, the geological data, and our analysis all yield similar results.

Critically, our results are significantly different from previous estimates of elevation change for WAIS Divide that rely on the implicit assumption that in the absence of elevation change, temperature change across Antarctica would be uniform (e.g., refs. 6 and 7), and which conclude that elevations may have been in excess of 300 m higher (and perhaps as much as 560 m higher) during the LGM than during the Holocene. Our analysis, in contrast, not only yields appreciably smaller elevation changes, but also suggests significantly different temporal changes.

Further insight on West Antarctic elevation change can be gained by comparison with numerical ice-sheet models. Albrecht et al. (52, 53) conducted an ensemble of experiments with the Parallel Ice Sheet Model (PISM) to evaluate the influence of climate forcing and model-parameter choices on the simulation of both the East and West Antarctic ice sheets over glacial-interglacial cycles. Importantly, they included the empirical snow-accumulation history from the WAIS Divide core (54) as part of the climate boundary conditions in a subset of their experiments. Like most such model experiments (e.g., ref. 55), they otherwise used accumulation forcing derived from the imposed temperature history (generally, the mean of East Antarctic temperature remonstrations), following a Clausius–Clapeyron-based scaling. With the EAIS scaling, their model output yields the relative elevation history shown by the dashed black line in Fig. 5. However, when using the empirically derived WAIS snow-accumulation (and separately, temperature) forcing, their experiments yield the elevation variations shown by the solid black line. The latter results show remarkably good agreement with our reconstructed changes in WAIS Divide surface elevation, produced through entirely independent methods.

These PISM simulations (52, 53) are the only published large-scale ice sheet modeling experiments that have used the empirical snow accumulation history from WAIS Divide as forcing. This comparison demonstrates that known climate and ice-sheet dynamics (as expressed through the ice-sheet model physics) are compatible with our results, and that the pattern of temperature variability in our reconstructions does not require especially large elevation changes. In that context, we note the extremely high LGM ice elevations reconstructed from glacier-isostatic modeling (e.g., refs. 56 and 57 “ICE6g” estimate, red line in Fig. 5), which are commonly used as boundary conditions for general circulation model (GCM) simulations of the LGM, such as those from the Paleoclimate Model Intercomparison Project (PMIP) (58). The ICE6g reconstruction shows elevation changes greater than 600 m at WAIS Divide and in excess of 2,000 m at locations within a few hundred km of the site (56, 57). Such large imposed elevation changes would tend to obscure the influence of other forgings and feedbacks, and the GHE-driven temperature-change pattern we have identified. In Section 5, *Materials and Methods* and *SI Appendix*, Fig. S15, we show that an independent estimate of LGM-to-Holocene temperature change (59), which uses a data-assimilation approach to address the limitations of unconstrained GCM simulations, does capture the temperature-dependent patterns expected from the GHE feedback. This reconstruction, the last glacial maximum reanalysis (LGMR), is consistent with our reconstructed pattern of warming, but not with the modeled temperature response (e.g., ref. 58) to very large imposed ice-sheet elevation changes [e.g., of ICE6g (56)].

## 4. Conclusions

Our results demonstrate that there is a fundamental pattern of temperature change in Antarctica, in which its magnitude is a function of the local mean surface temperature. This is the consequence of the temperature dependence of the greenhouse effect, which is nonlinear at the cold temperatures characteristic of the Antarctic ice sheet. The temperature dependence of the thermodynamic processes driving the nonlinearity in GHE, in particular the water-vapor and lapse-rate feedbacks, is supported by observations, a convective-radiative equilibrium model, and an atmospheric GCM. Its effects are revealed by a systematic analysis of ice core data across a range of timescales.

At the longest timescales associated with Milankovitch orbital forcing, the GHE feedback explains nearly all of the differences in the magnitude of temperature variability between different locations in Antarctica. Across a wide range of shorter (millennial) timescales, the same feedback still explains the majority of the observed spatial pattern. The simplicity and timescale independence are critical features of the observed pattern. They indicate that the response is independent of the nature of the initial forcing. Instead they suggest a fast, temperature-dependent feedback amplifying a forcing. If the observed temperature-change pattern were to arise from a slow feedback, such as changes in ocean circulation (that cause changes in poleward heat transport) or changes in size of the Antarctic ice sheets, lags would be observed compared to the mean change across different timescales and the emerging pattern would not be a simple function of initial surface temperature.

Deviations from the pattern arising from the GHE feedback are expected if the pattern of forcing is not uniform, and if spatial variability in the forcing is large compared to the continent-wide mean forcing. The largest deviations from the mean pattern can be explained by ice-sheet elevation changes, particularly as observed in West Antarctica between the Last Glacial Maximum and the Holocene. Importantly, the implied elevation change is smaller than some previous estimates suggest, and the timing is different, but is consistent with ice-sheet model simulations.

Over most of the Earth, radiation to space increases with surface temperature at a rate much less than given by the Stefan–Boltzmann equation, owing to the greenhouse effect. However, at the coldest Antarctic temperatures, as the GHE is severely diminished, outgoing radiation approaches that of a blackbody. The highest, coldest parts of Antarctica can satisfy a change in energy balance with a small change in temperature, while a larger temperature change is required at lower, warmer regions of the continent owing to the radiatively more opaque atmosphere. The transition between these two regimes occurs uniquely across the range of Antarctic annual mean surface temperatures, concomitant with the strong nonlinearity of the GHE. Antarctica is our planet’s Southern radiator. The efficiency of its response to an energetic forcing depends on the surface temperature and leads to a fundamental pattern of Antarctic temperature change.

## 5. Materials and Methods

### 5.1. Ice Core Water Isotope Records.

*SI Appendix*, Fig. S1 shows the raw water isotope records used for the temperature reconstructions shown in Fig. 1. The records include WAIS Divide [WDC, (13, 33, 38)], Siple Dome (60, 61), EDML (62), EDC (62, 63), Vostok (64), Dome Fuji (65), Talos Dome (66), and South Pole [SP, (67)]. Where available, records are on age scales synchronized by refs. (7, 31) to the WD2014 age scale (68) (WDC, Talos Dome, Dome Fuji, EDML, EDC, Siple). At ages older than the limit of that synchronization, we use the AICC23 age scale (32) for Talos Dome, EDML, EDC, as well as for the Vostok record. We otherwise use the most recent published age scale for each core. We employ the temperature reconstruction technique described in Markle and Steig (4), which makes use of the simple water isotope model (SWIM) and requires measurements of both $\delta^{18}$O and δD. We apply the technique to somewhat longer records from EDC and Vostok than presented in that study. By using both $\delta^{18}$O and δD data, and the nonlinear framework of SWIM, we correct the surface temperature reconstructions for coincident changes in moisture source evaporation conditions that may also influence water isotope ratios of Antarctic precipitation. We use the full uncertainty estimation from ref. 4, which assessed the influence of model parameterizations and other choices including fractionation factors, the evaporation closure assumption, the supersaturation parameterization, assumptions about precipitation, and the influence of vapor mixing during transport, among others, outlined extensively in ref. 4. To account for the possibility of systematic changes in the relationship between surface temperature and vertically integrated condensation temperature, we make a more conservative estimate of the uncertainty in this relationship than in ref. 4, using a slope of 0.69±0.07.

The essential pattern of reconstructed surface temperature change from the LGM to Holocene, shown in Fig. 3*A*, is also seen in the raw water isotope records. The initial $\delta^{18}$O is a strong predictor of total LGM-Holocene $\delta^{18}$O change (*SI Appendix*, Fig. S2*A*). We include the Byrd $\delta^{18}$O record in this analysis (69, 70); it was not included in the temperature reconstructions owing to the lack of concurrent δD data of sufficient precision. Because the averaging blocks are large (0 to 10 ka and 20 to 30 ka), uncertainties in the older Byrd age scale do not substantially impact the results.

In our reconstructions of surface temperature, changes in moisture-source temperatures for each core site, shown in *SI Appendix*, Fig. S2*B*, are accounted for. The only substantial difference in the pattern of $\delta^{18}$O change between LGM and Holocene compared to the equivalent pattern for reconstructed surface temperature (Fig. 3) is at the Talos Dome site, for which the combination of $\delta^{18}$O and δD data indicate larger moisture source temperature change compared to other sites. Such anomalous changes reduce the amplitude of local $\delta^{18}$O change relative to other sites (4).

### 5.2. The Greenhouse Effect.

*SI Appendix*, Fig. S4*A* shows the upwelling longwave radiation at the surface (black dots, LW_*s*_) as well as the outgoing longwave radiation at the top of the atmosphere (red dots, OLR) as a function of latitude, and their difference, the GHE (blue dots). The complexity in this spatial pattern collapses when we examine the same data as a function of surface temperature (*SI Appendix*, Fig. S4*B*). The longwave emitted from the surface is calculated assuming a surface emissivity, ϵ, of 0.99. Typical emissivities of snow range from 0.98 to 0.99 for conditions relevant for the ice-core sites (16), though can be as low as 0.93 for coarse-grained snow. Assumed surface emissivities within this range do not qualitatively change our results.

We calculate a second-order polynomial fit of GHE to surface temperature from the NCEP/NCAR reanalysis (17) and AIRS observational data (18, 19), at both monthly and annual average time scales (*SI Appendix*, Fig. S4 *C* and *D*). Because the AIRS data are observational, higher resolution, and extend to lower surface temperatures, we prefer them over the NCEP/NCAR reanalysis for estimating the temperature dependence of the GHE, and similarly because the monthly AIRS data cover a broader temperature range than the annual average data, we use them for our default calculations in the main text. The monthly and annual mean AIRS fits are marginally different as shown in *SI Appendix*, Fig. S4*D*. The coefficient of determination for the fit to monthly data is $R^{2}=0.93$ and $R^{2}>0.99$ for the fit to annual mean data. We use the difference between the monthly and annual mean fits (and their 95% confidence bounds) as an estimate of the uncertainty in the GHE prediction in Fig. 4 in the main text. The feedback is calculated as the temperature derivative of these fits, $c\left(T\right)=\frac{\mathrm{dGHE}}{\mathrm{dT}}$.

We show the range of $\lambda_{0}$, the feedback c(T), and feedback factor $f=\lambda_{0}c$, as a function of Antarctic surface temperature in *SI Appendix*, Fig. S5, as well as the spread resulting from the uncertainty in the GHE fits. The system gain,

[4] $$\begin{array}{c} \frac{\Delta T}{\Delta T_{0}}=\frac{1}{\left(1-f\right)}=\frac{1}{1-c\lambda_{0}}, \end{array}$$

quantifies the final temperature change resulting from the GHE feedback, compared to the reference temperature response, $\Delta T_{0}=\lambda_{0}\Delta F$ (*SI Appendix*, Fig. S5*D*).

The feedback c(T) is diagnosed from

[5] $$\begin{array}{c} c\left(T\right)=\frac{d}{\mathrm{dT}}GHE=\frac{d}{\mathrm{dT}}\left(LW_{s}-OLR\right). \end{array}$$

Because the longwave emission from the surface follows Eq. **1**, the derivative of $LW_{s}$ with respect to surface temperature is

[6] $$\begin{array}{c} \frac{d}{\mathrm{dT}}LW_{s}=4\epsilon \sigma T^{3}=\frac{1}{\lambda_{0}}. \end{array}$$

We can rewrite c(T) as

[7] $$\begin{array}{c} c\left(T\right)=\frac{1}{\lambda_{0}}-\frac{d}{\mathrm{dT}}OLR. \end{array}$$

Substitution into Eq. **3** and simplification leads to

[8] $$\begin{array}{c} \Delta T=\frac{\Delta F}{\frac{d}{\mathrm{dT}}OLR}. \end{array}$$

The relevant nonlinearity is thus captured by the observed surface-temperature dependence of the OLR. The essential feature is that over the range of Antarctic surface temperatures (Fig. 2 *A* and *B*) the GHE, and therefore the OLR, transitions from being a strongly nonlinear function of the surface temperature to being a nearly linear one. Thus while the derivative with respect to temperature, and therefore c(T), may be constant at warmer temperatures, it is a function of surface temperature across Antarctica. Note that the calculation of the response pattern (Eq. **8**) does not depend on the assumed emissivity of the surface, though the system gain (Eq. **4**) does.

We use the MIT single-column radiative-convective equilibrium model to investigate the robustness of the nonlinearity in the greenhouse effect. The model is a one dimensional, full spectrum radiative transfer and heat convection model that accounts for cumulus and layer clouds (20–23). We run the model through a range of incoming shortwave insolation values at TOA, representative of latitudes from 90^°^ S to the Equator. The model solves for, among other things, the profiles of atmospheric temperature (*SI Appendix*, Fig. S6*A*) and water vapor, as well as the surface and TOA outgoing longwave radiation, from which we calculate the greenhouse effect (*SI Appendix*, Fig. S6*B*). Testing a large range of model parameter choices (including surface ocean fraction, prescribed vs. dynamic surface albedo, surface emissivity, the background concentration of other greenhouse gases besides water vapor) leads to qualitatively similar results. The nonlinearity in GHE occurs where the OLR and LW_*s*_ curves converge, and where the Earth transitions from radiating longwave to space like a blackbody, to having to radiate energy through an opaque atmosphere. Because this is a column model which lacks horizontal atmospheric heat transport (which warms the cold high latitudes at the expense of the warm low latitudes), the range of surface temperatures resulting from the model is much broader than on the real planet. Despite this, the convergence of the OLR and LW_*s*_ curves, and thus the characteristic nonlinearity and magnitude of the GHE, occurs over the same range of Antarctic surface temperatures in both the model and observations (*SI Appendix*, Fig. S6*B*). This demonstrates that the nonlinearity of the GHE is a fundamental result of thermodynamic processes in the atmospheric column, is a robust phenomenon at Antarctic surface temperatures, and does not depend on atmospheric heat transport.

The individual processes driving the greenhouse effect, e.g., water vapor, the free atmosphere lapse rate, and some shortwave processes, are themselves individual feedbacks, though the impacts of water vapor and the lapse rate are often combined (8). We compare the GHE feedback calculated from observations to the sum of individual feedbacks diagnosed from the Community Atmosphere Model 5 (CAM5) using the kernel method (24). We plot the sum of the water-vapor, lapse-rate, and shortwave feedbacks in CAM5 as a function of the surface temperature at every grid point over the Antarctic continent (*SI Appendix*, Fig. S7). The change in the magnitude of these feedbacks with surface temperature is in good agreement with the simple calculation of the temperature dependence of the observed $\frac{\mathrm{dGHE}}{\mathrm{dT}}$. The change in the greenhouse effect over Antarctica is not just the water-vapor feedback, or the lapse-rate feedback, or shortwave feedbacks acting in isolation, but rather the sum of these processes.

### 5.3. Principal Component/Empirical Orthogonal Function Analysis.

In *SI Appendix*, Fig. S8 we show the results of the principal component/empirical orthogonal function (PC/EOF) analysis. *SI Appendix*, Fig. S8 *A* and *B* show the scores of all PCs, while panel *C* shows the loadings of each PC at all sites (the EOFs), and panel *D* shows just the loadings for PC1 at each site. Panel *E* shows the variance explained by each PC; PC1 explains the vast majority of the variance across sites.

### 5.4. Pattern Across Sites for Intervals of Warming and Cooling.

To assess the pattern of temperature change across all timescales, we perform the sliding block analysis, as described in the main text, on both the raw temperature reconstructions and on the temperature expression of PC1. This results in analyzing the pattern of temperature change for $1.35\times 10^{4}$ different intervals on the temperature reconstructions and $9.7\times 10^{3}$ intervals on PC1. We perform the analysis back to 70 ka on the raw records, which includes intervals with fewer than eight total records. We perform the analysis back to 54 ka on PC1, as this is the period of overlap for all eight records. It is hard to see the individual results of ≈10^4^ different and overlapping intervals of change due to the large number of iterations of the analysis, so we only show every 5th line of best fit in Fig. 4. In *SI Appendix*, Fig. S9, we provide visualizations of this analysis for both the temperature reconstructions and PC1, showing every 50th (panels *A* and *B*), every 10th (panels *C* and *D*), and all intervals analyzed ($1.35\times 10^{4}$ in panel *E* and $9.7\times 10^{3}$ in panel *F*). This allows the reader to better see how the individual lines of best fit of the pattern align with the prediction from GHE (black lines in all panels). As can be seen in the panels of *SI Appendix*, Fig. S9, repeating the sliding block analysis on PC1 removes the noise associated with random intervals, particularly when the mean change in temperature is small. The resulting cleaner pattern is well predicted by the GHE.

The essential pattern revealed in the sliding block analysis is robust to the temperature reconstruction technique. In *SI Appendix*, Fig. S10 we show the result of the analysis performed on the temperature reconstructions compared to the same analysis on the raw $\delta^{18}$O data.

The feedback associated with the GHE predicts that during intervals of continent wide warming the warmest parts of the continent should warm more than the coldest parts. Conversely, it predicts that during intervals of continent wide cooling the warmest parts of the continent should cool more. As described in the main text, this suggest that the slopes to the lines of best fit for the change at all sites as a function of the initial temperature at all sites, $\frac{d(\Delta T)}{dT_{i}}$, should be greater than zero during intervals when $\bar{\Delta T}>0$ (mean warming), and conversely $\frac{d(\Delta T)}{dT_{i}}$ should be less than zero when $\bar{\Delta }T<0$ (mean cooling). This statement alone does not predict the magnitude of $\frac{d(\Delta T)}{dT_{i}}$, only its sign during intervals of either warming or cooling. In *SI Appendix*, Fig. S11 we show the histograms of $\frac{d(\Delta T)}{dT_{i}}$ for all lines of best fit to all $1.35\times 10^{4}$ intervals examined in the sliding block analysis described in the main text. For intervals of continent-wide warming, the distribution of $\frac{d(\Delta T)}{dT_{i}}$ is persistently positive, while $\frac{d(\Delta T)}{dT_{i}}$ is consistently negative during intervals of continent wide cooling. The means of these distributions of slopes are significantly different than zero (P<0.01) for either warming or cooling. The pattern is even more pronounced if we consider only the reconstructions on synchronized age scales, or only intervals when the mean Antarctic temperature change is significantly different from zero. Further, this patterns holds if we restrict our analysis only to records from the EAIS. In all cases, the mean of the distribution of slopes is significantly different from zero.

This distinction in the pattern between sites is perfectly divided if we examine the histogram of slopes resulting from the sliding block analysis performed on the projection of PC1 at all sites (*SI Appendix*, Fig. S12). There are no intervals of warming in which $\frac{d(\Delta T)}{dT_{i}}<0$ and no intervals of cooling in which $\frac{d(\Delta T)}{dT_{i}}>0$ for the temperature expression in PC1. Of course this result is intuitive once the loading of EOF1 across sites is known.

The fact that the sign of the pattern among sites is predicted by the sign of the mean temperature change across all sites, is a first-order test of the GHE hypothesis. This relationship is predicted by the GHE and opposite to the relationship predicted by the Planck response alone. It is also inconsistent with uniform temperature change being the common pattern across the continent. Further, the GHE hypothesis predicts that not only is the sign of the relationship between sites predicted by the sign of the mean change, but the magnitude of $\frac{d(\Delta T)}{dT_{i}}$ is predicted by the magnitude of $\bar{\Delta T}$ (Fig. 4 *E* and *F*).

### 5.5. The Last Four Glacial Cycles.

Four records in our compilation extend to the last interglacial period: EDML, EDC, Fuji, and Vostok, with the latter three further covering the last four glacial cycles to 400 ka. The initial site temperature is a strong predictor of the warming during the penultimate deglaciation (*SI Appendix*, Fig. S3). This holds for the previous two glacial cycles as well, though the result is inherently noisier with only three records. We can partly overcome this limitation by investigating many distinct time intervals using the sliding block analysis described in the main text. Here, we use block sizes x of 4 to 20 ka in steps dx= 4 ka, a separation y from 10 to 100 ka, in steps of dy= 5 ka. We slide these blocks across the time interval 0 to 400 ka, in steps of dt= 10 ka. We thus evaluate nearly 4,000 intervals of change in the $\delta^{18}$O, temperature reconstructions, and PC1 of the temperature reconstructions. The results (*SI Appendix*, Fig. S13) confirm the findings from the reconstructions covering only the last glacial period: ΔT is a clear function of initial surface temperature.

### 5.6. Elevation Changes.

*SI Appendix*, Fig. S14 shows the sensitivity of our reconstruction of elevation changes at WAIS Divide to how we assess anomalous temperature variability (panel *A*) and to our assumption of the surface lapse rate (panel *B*). Superadiabatic surface lapse rates (e.g., $\gamma =14^{{}^{\circ}C}/km$) are typical of the modern high East Antarctic plateau (48) and lead to smaller estimated elevation changes at WAIS Divide. Subadiabatic lapse rates (e.g., $\gamma =8^{{}^{\circ}C}/km$) are only typical of the coastal escarpment regions of the continent (48), and are not likely relevant for the WAIS Divide site. The adiabatic and superadiabatic estimates thus represent a likely range of variability.

Ice sheet model simulations such as those shown in Fig. 5 are quite sensitive to unknown parameters. In particular, the assumed basal traction parameter (in PISM, this is parameterized as the “critical till angle,” ϕ) has an influence on the magnitude and duration of the accumulation-driven peak in ice-sheet elevation (52, 53). The results shown in Fig. 5 assume $\phi =3^{{}^{\circ}}$. Use of other values within the range considered plausible change the magnitude and the timing of the peak elevation changes at WDC as shown in *SI Appendix*, Fig. S14, but do not alter the general agreement of the PISM simulations forced by West Antarctic accumulation with our independent results.

### 5.7. The LGMR.

The GHE feedback may help to explain the pattern of Antarctic temperature change in other, independent reconstructions. For example, the LGMR (59) used simulated priors from the community Earth system model (CESM), paleoclimate proxy records, and data assimilation techniques to reconstruct the warming since the last glacial maximum. Importantly this reconstruction did not include Antarctic ice cores in its observations (only in validation), yet the pattern of relative warming is largely consistent with our reconstructed pattern of relative warming (*SI Appendix*, Fig. S15). As the reanalysis did not include observations from the continent, it may be particularly sensitive to the model simulation priors, and the imposed forcing on those simulations. These imposed forcings include ice-sheet elevation changes from ICE6G (56), which are significantly larger than our findings for WAIS Divide. The CESM priors are very strongly influenced by the prescribed forcing of the ice sheet changes, with local temperature changes over Antarctica that are very large compared to our ice core reconstructions. However, the LGMR does not impose the ICE6G elevations on the final reconstruction; rather, the elevation history is treated as part of the uncertainty (as in, e.g., ref. 71). The LGMR results show that the initial surface temperature is a strong predictor of the total warming across the continent, in good agreement with our reconstructions. The GHE feedback predicts the pattern of the dependence of total warming on initial surface temperature well, and the variance can be explained by spatial variability of about 5 Wm^−2^ in the mean forcing (*SI Appendix*, Fig. S15). This result is important in that it shows that even if there is regionally variable forcing around a central mean (e.g., due to locally variable changes in ice sheet height, atmospheric transport, or spatial patterns in insolation) the GHE feedback can explain the overall pattern of warming. Neither the LGMR nor our reconstruction are consistent with the very large changes in ice sheet height suggested by the ICE6G reconstruction (despite that these force the prior of the LGMR).

## Supplementary Material

Appendix 01 (PDF)

## Data Availability

All ice core reconstructions and underlying data are available at https://doi.org/10.5281/zenodo.19009077 (72). This study uses previously published reconstructions (4) and ice-core water-isotope datasets. The WAIS Divide dataset (38) is available at https://dx.doi.org/10.17911/S9MW2F and the South Pole dataset (67) is at https://dx.doi.org/10.15784/601239. The other ice-core records are available from the original publications (see text and refs. 62–66). This work uses NCEP/NCAR reanalysis data (17), AIRS data (17–19), feedback estimates from CAM5 (24), and LGMR data (59). The ice sheet reconstructions are available from the original publications (see text and refs. 52, 53, and 56).
