# Supplementary material for: Temperature-dependent feedbacks drive the pattern of Antarctic temperature change
Source: Proc Natl Acad Sci U S A. 2026 May 11;123(20):e2513383123. doi: 10.1073/pnas.2513383123 (PMC13187729; doi:10.1073/pnas.2513383123)
Supplement: Supplementary file 1 — Appendix 01 (PDF) [file pnas.2513383123.sapp.pdf]

PNAS

Supporting Information for

Temperature-dependent feedbacks drive the pattern of Antarctic temperature change

Bradley R. Markle, Eric J. Steig

Bradley R. Markle.
E-mail: bradley.markle@colorado.edu

This PDF file includes:

Figs. S1 to S15
SI References

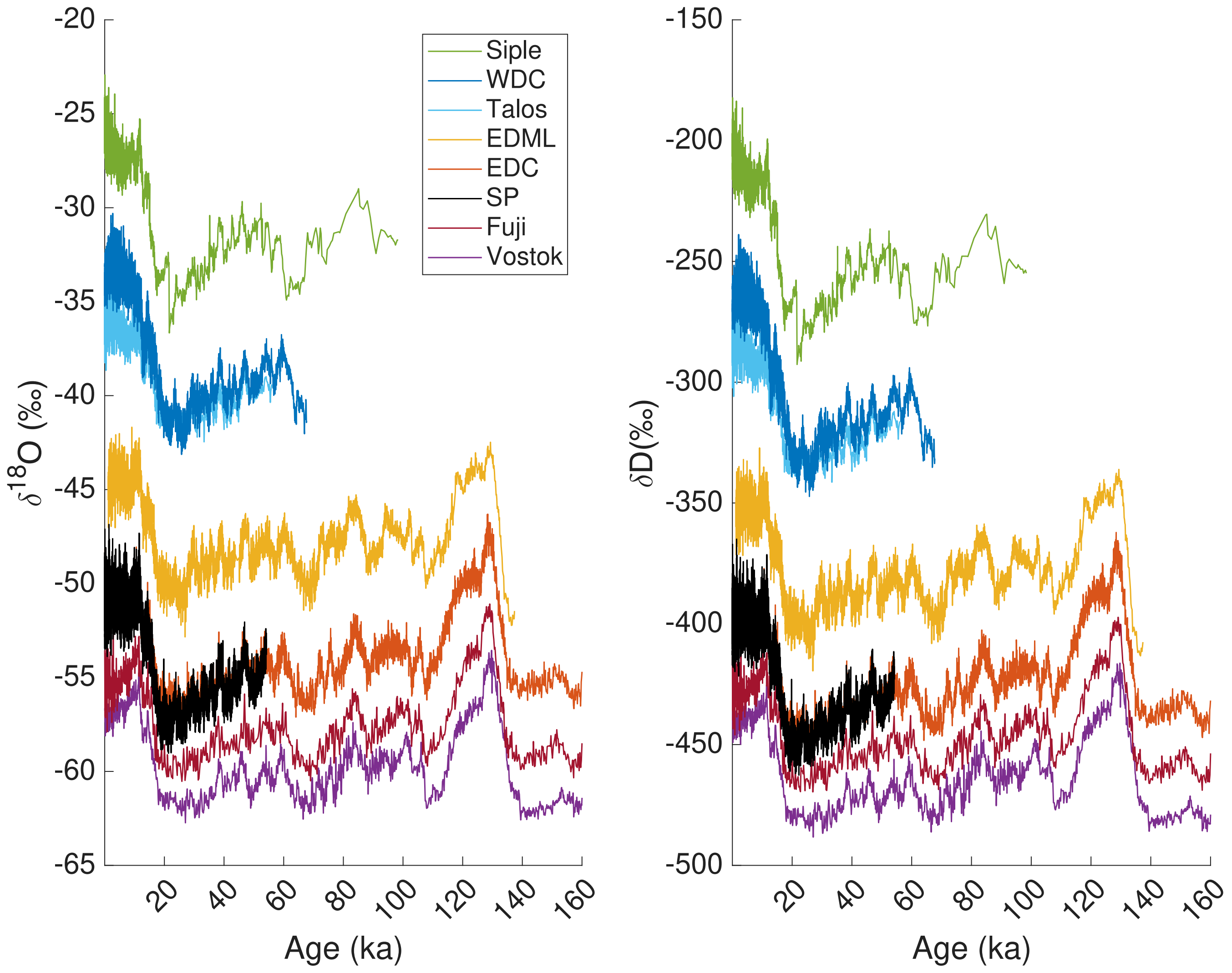

Fig. S1. Records of δ^{18}O and δD from deep Antarctic ice cores. The records include WDC (1–3), Siple Dome (4, 5), EDML (6), EDC (6, 7), Vostok (8), Dome Fuji (9), Talos Dome (10), and South Pole (SP, (11)).

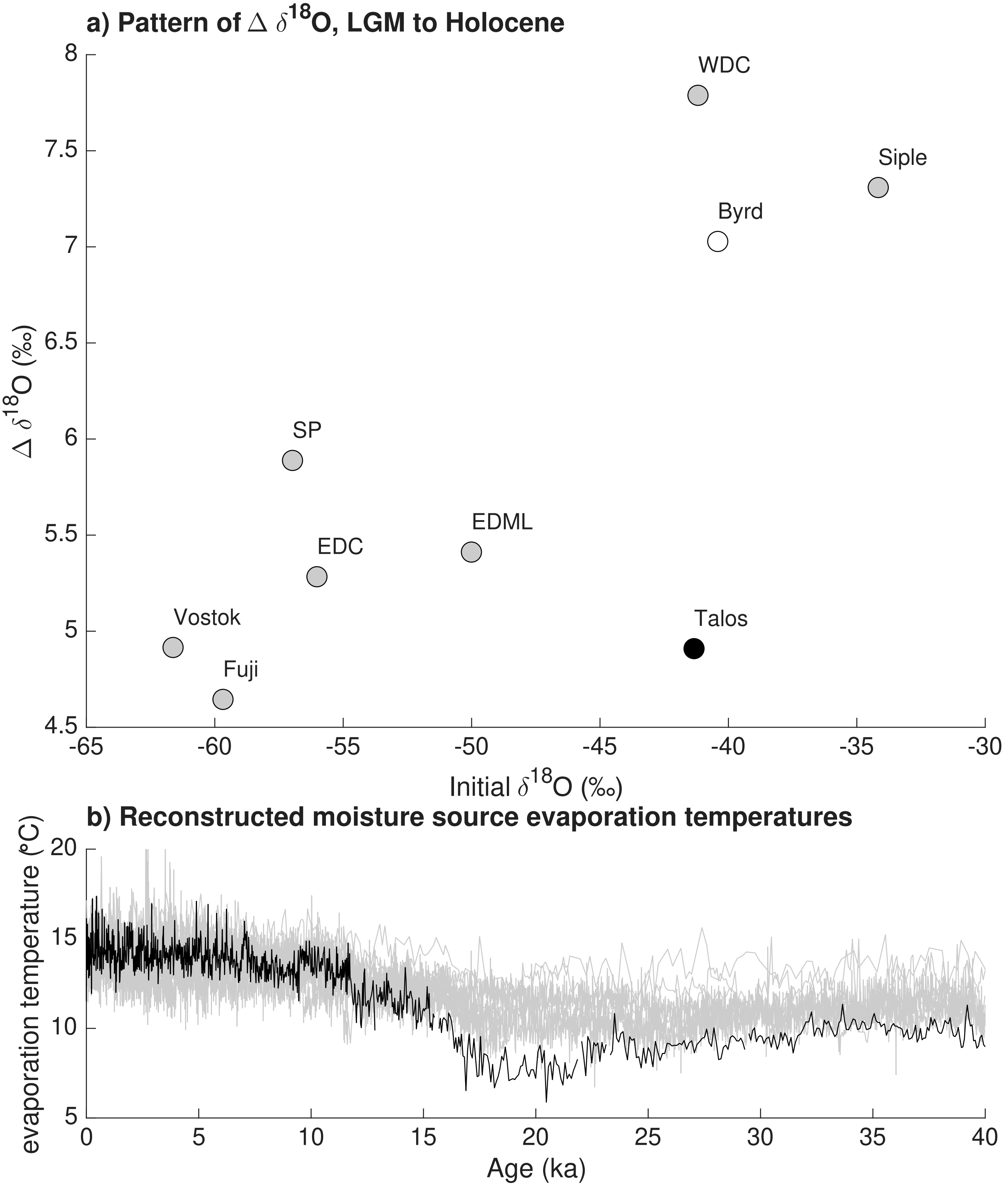

Fig. S2. a) The pattern of LGM-Holocene change in the δ^{18}O records as a function of initial δ^{18}O. **b)** The reconstructed changes in moisture source temperatures for each core site (12). All cores are shown in grey except for Talos Dome, shown in black.

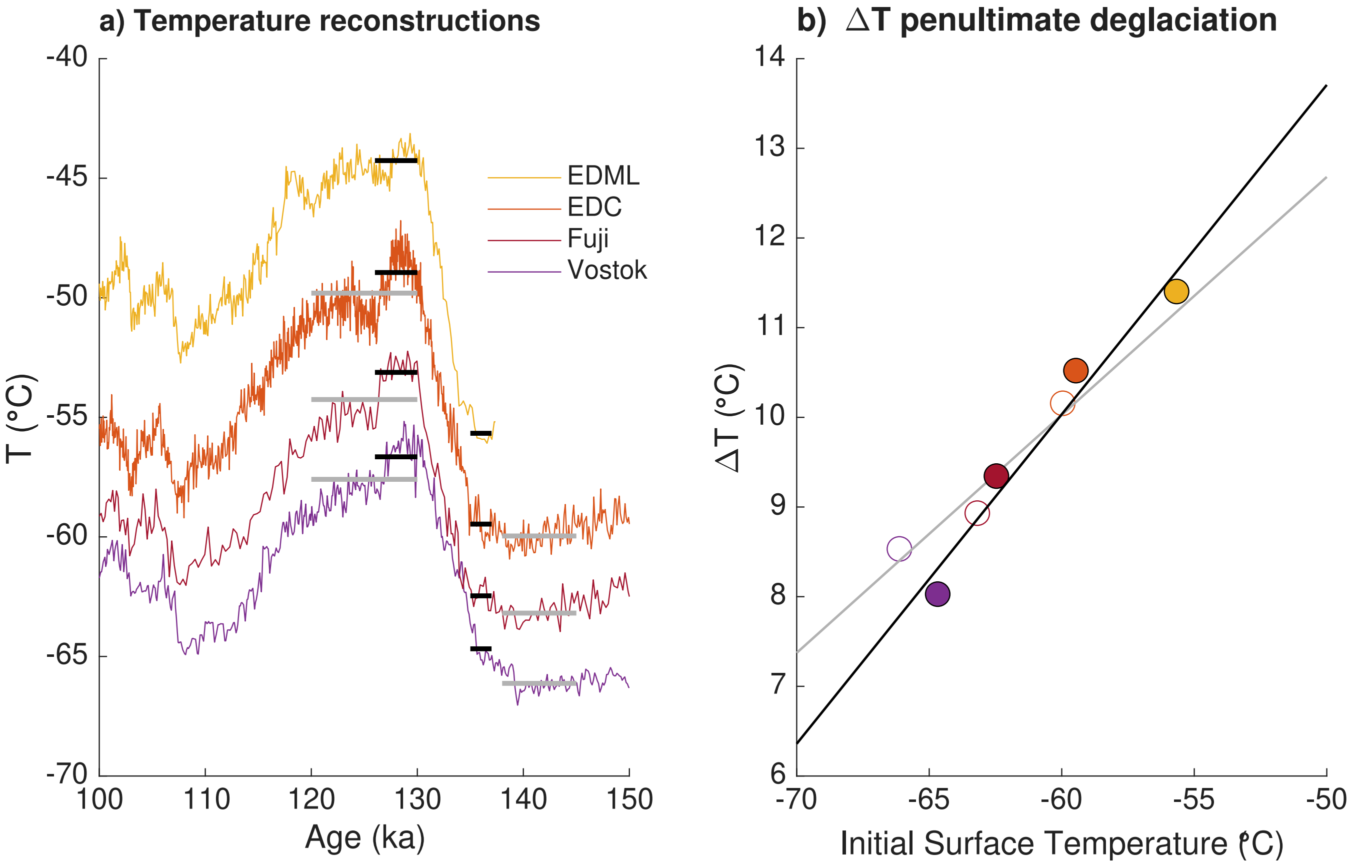

Fig. S3. a) Temperature reconstructions for the EDML, EDC, Fuji, and Vostok records over the last interglacial period and penultimate deglaciation. **b)** The pattern of temperature change over the penultimate deglaciation. Filled circles show the warming, across four records, between the time intervals indicated by the black bars in panel **a**; black line shows a linear fit. Open circles, with grey line of best fit, show the warming between longer baseline intervals, indicated by the grey bars in panel **a**, but for which only three cores are available.

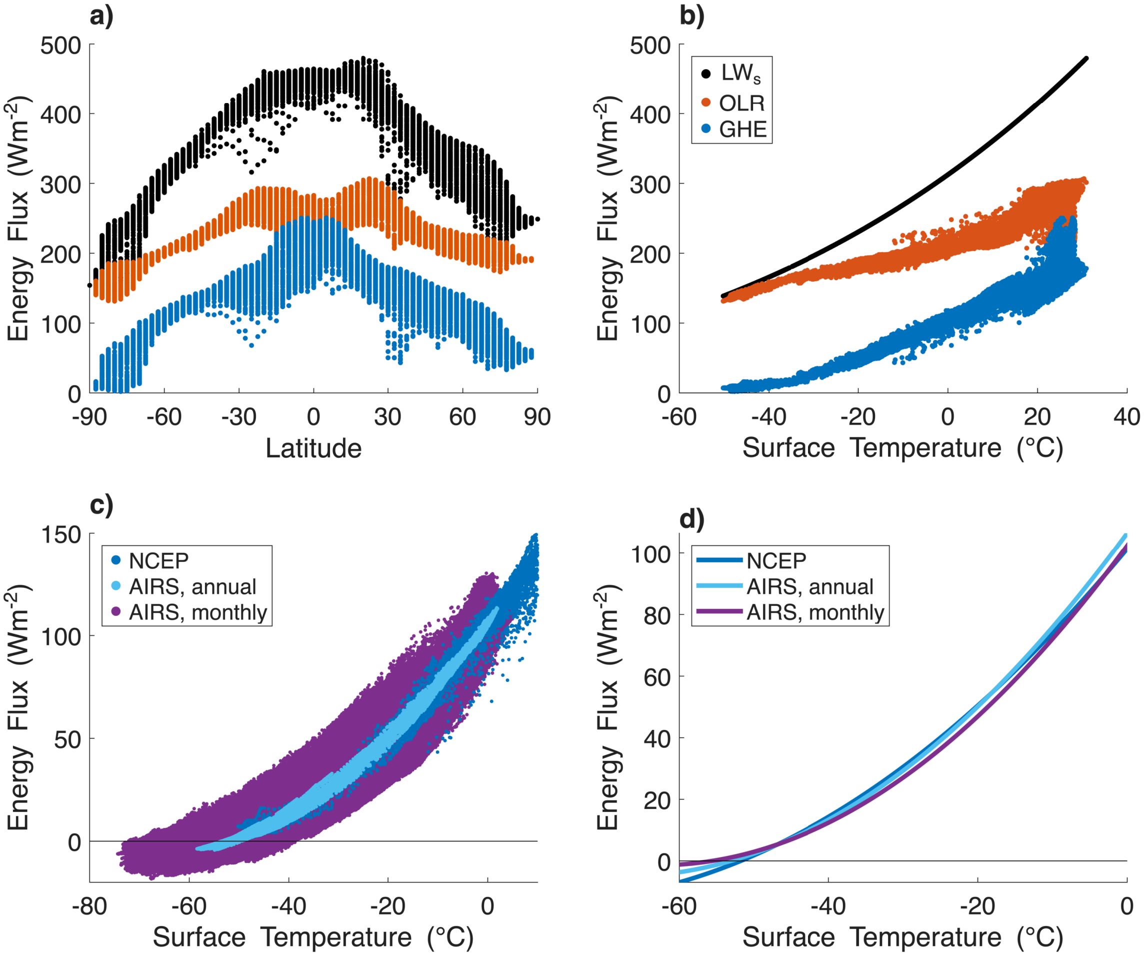

Fig. S4. **a)** Upwelling longwave radiation at the surface (black dots), which is calculated from surface temperature, as well as the outgoing longwave radiation at the top of the atmosphere (red dots), and GHE (blue dots) from the NCEP/NCAR reanalysis (13). **b)** Same as in panel **a** but plotted as a function of surface temperature rather than latitude. **c)** The greenhouse effect plotted as a function of surface temperature from the NCEP/NCAR reanalysis (blue), annual mean AIRS data (cyan, (14, 15)), and monthly AIRS data (purple). **d)** Second order polynomial fit of GHE to surface temperature from the NCEP/NCAR data (blue), annual mean AIRS data (cyan), and monthly AIRS data (purple).

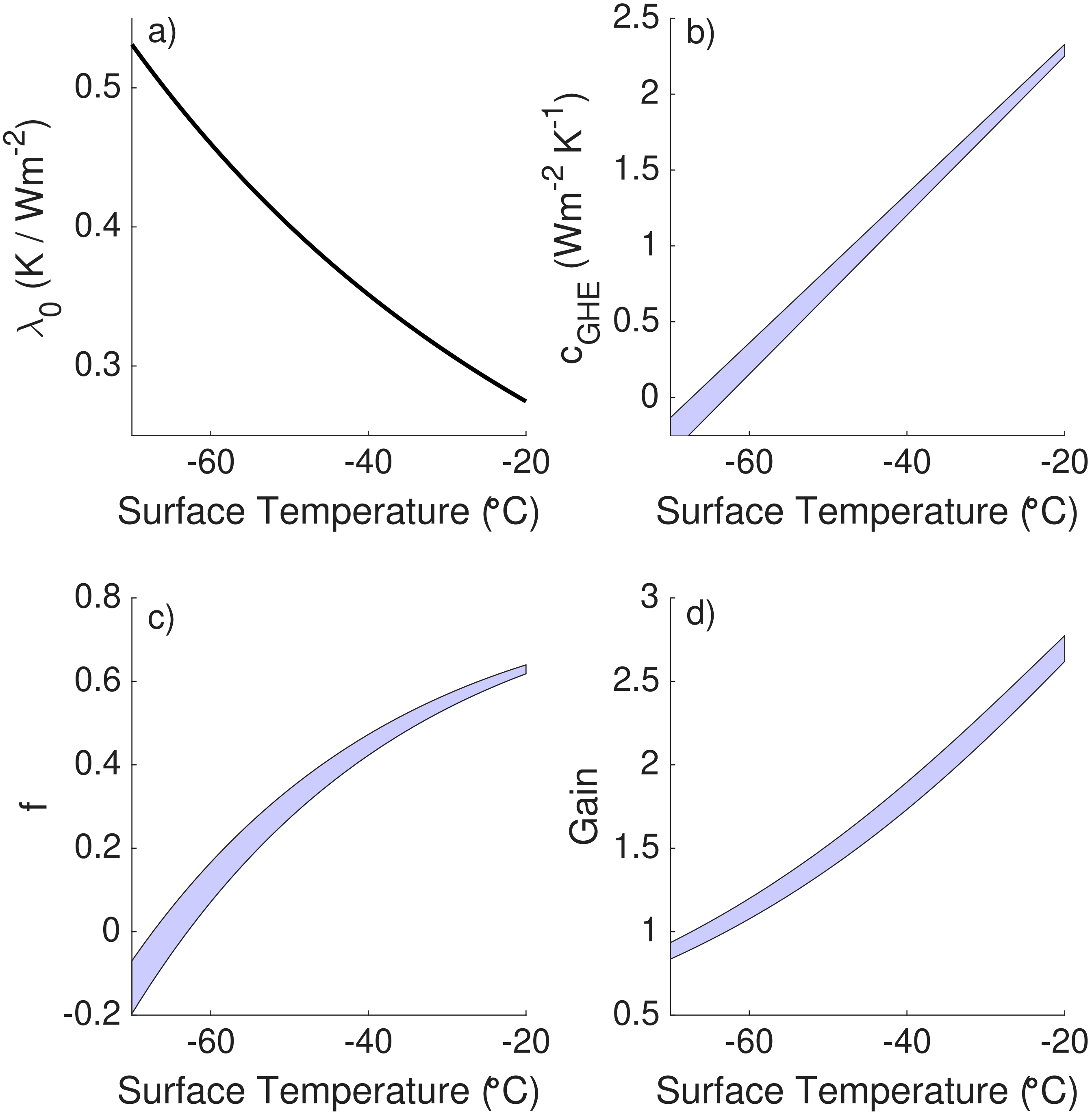

Fig. S5. Parameters related to the greenhouse effect (GHE). **a.** The reference system (Planck) response, λ_0, as a function of surface temperature. **b.** The feedback, c, as a function of temperature calculated as $c = \frac{dGHE}{dT}$, with the spread arising from the spread of fits to monthly and annual average AIRS data and their uncertainties. **c.** The feedback factor, $f = \lambda_0 c$, for the GHE feedback. **d.** The gain of the system, $\frac{\Delta T}{\Delta T_0} = \frac{1}{(1-f)}$.

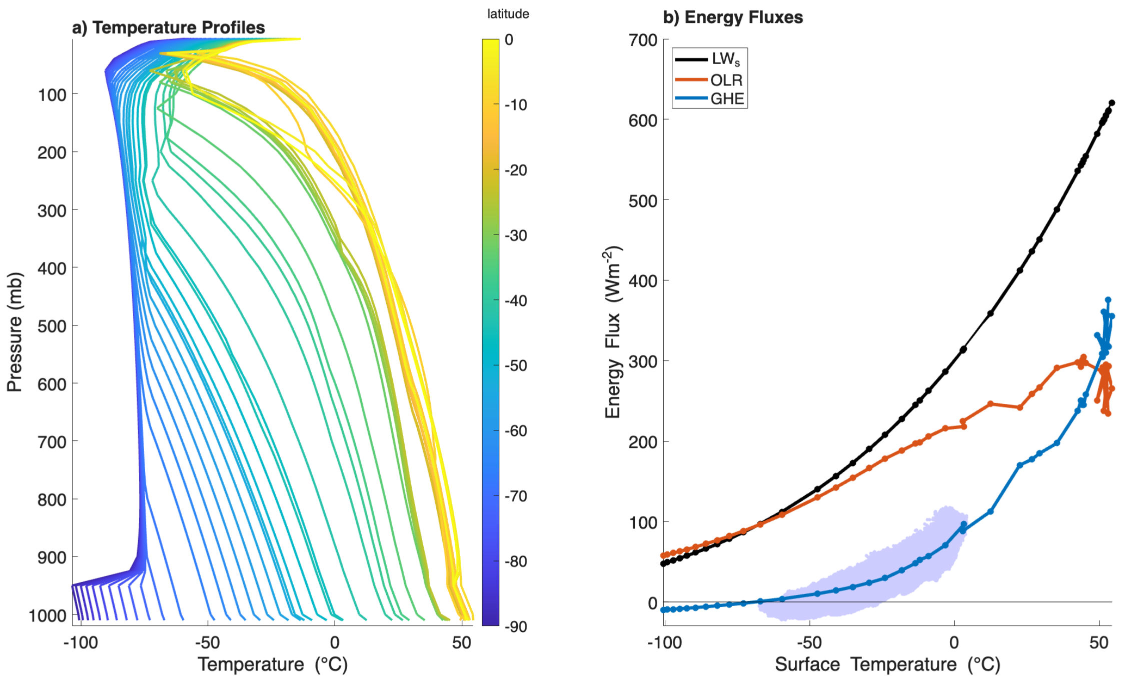

Fig. S6. a. Vertical temperature profiles from the Radiative Convective Equilibrium Model across all latitudes of the Southern Hemisphere (color scale) representing a range of TOA insolation. Note that the range of surface temperatures are not directly mappable to the real world's latitudes as the model lacks horizontal heat convergence. The GHE is a function of the vertical lapse rate, water vapor, and cloud albedo which are all represented in the model. **b.** The upwelling longwave radiation from the surface (black line, LW_s), OLR (red line), and GHE (blue line), as a function of surface temperature resulting from the model. While the range of surfaces temperatures is large compared to the real world, the nonlinearities of these curves as a function of surface temperature is extremely consistent with reality. The light blue dots show the GHE diagnosed from AIRS data.

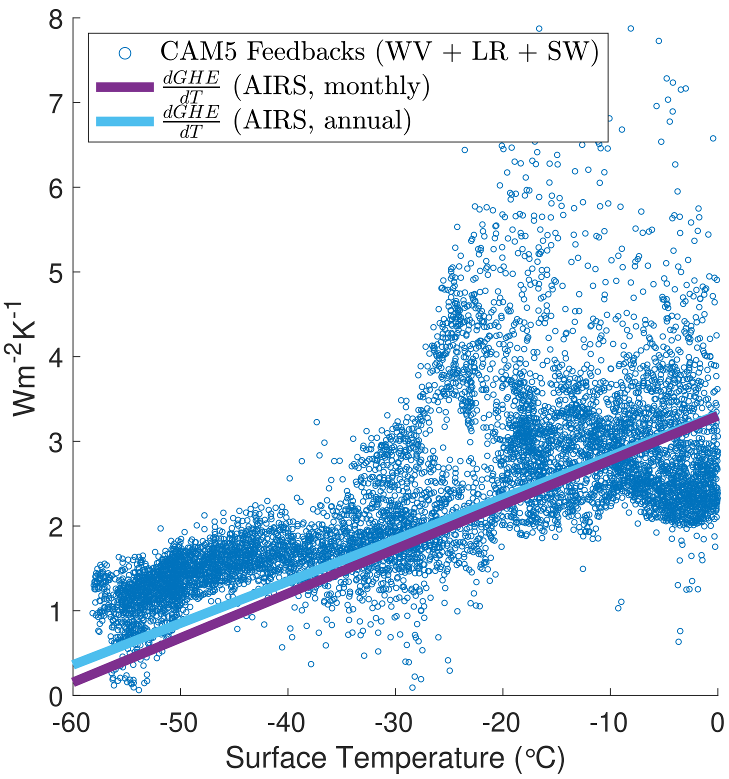

Fig. S7. The sum of the water-vapor (WV), lapse-rate (LR) and shortwave (SW) feedbacks at each Antarctic grid point in CAM5 as a function of surface temperature (16). Different estimates of the derivative of the greenhouse effect (GHE) with respect to surface temperature from the AIRS data (14, 15) are shown in colored lines. The purple line shows the derivative of a 2nd order polynomial fit to monthly AIRS data, while the cyan line shows the derivative of a 2nd order polynomial fit to mean climatology of the annual AIRS data.

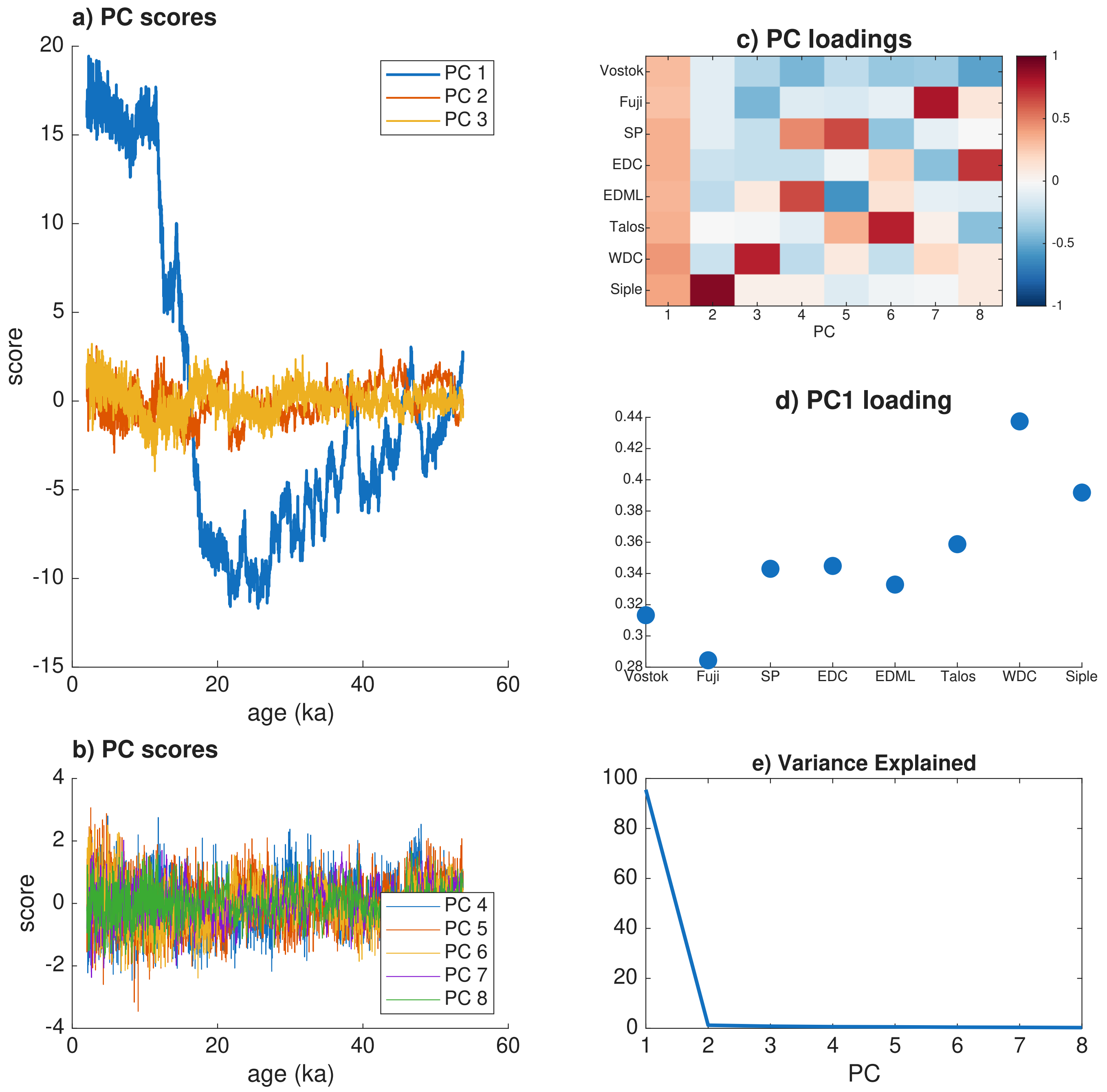

Fig. S8. a. The scores of the first three leading principal components (PCs). **b.** The scores of the remaining five PCs. **c.** The loading (or EOFs) of each core in each PC. **d.** The loading of each core in PC1, EOF1. Cores are arranged by modern surface temperature. **e.** The percent of total variance explained by each PC.

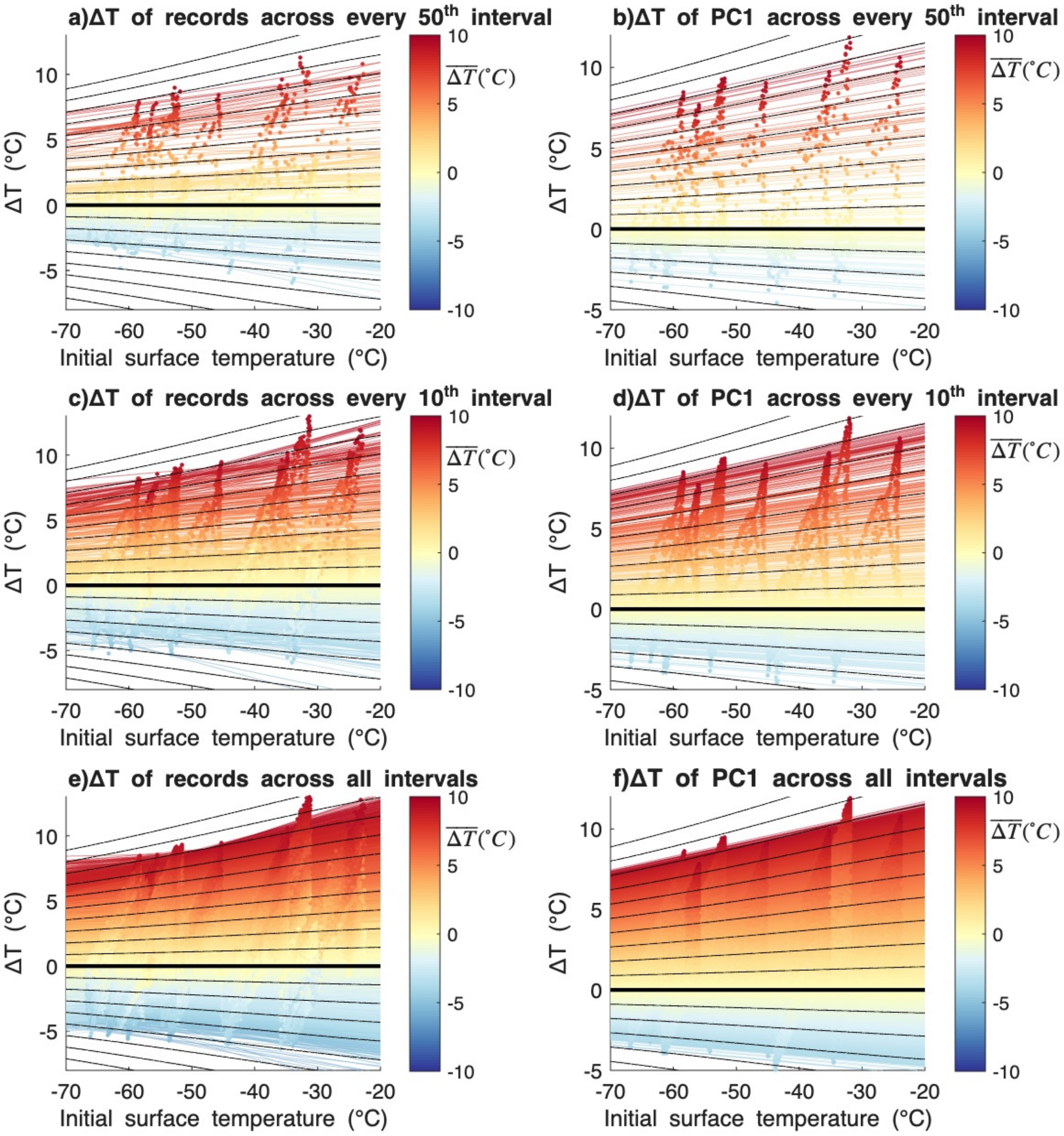

Fig. S9. The sliding block analysis, described in the main text, on both the reconstructions (left panels) and PC1 (right panels) in which only every 50th interval is shown (top row), every 10th interval is shown (middle row), and all intervals are shown (bottom row).

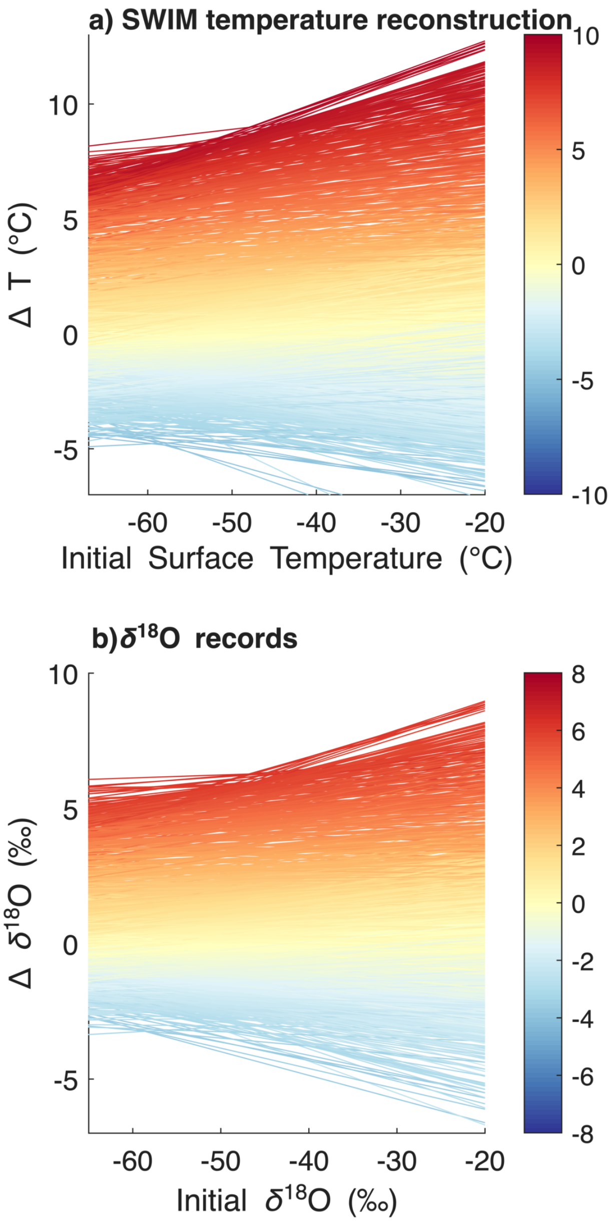

Fig. S10. The sliding block analysis, described in the main text, on **a)** the temperature reconstructions and on **b)** the raw δ^{18}O records, colored by the mean change of either temperature or δ^{18}O over the interval examined. Only every 5th interval is shown for clarity.

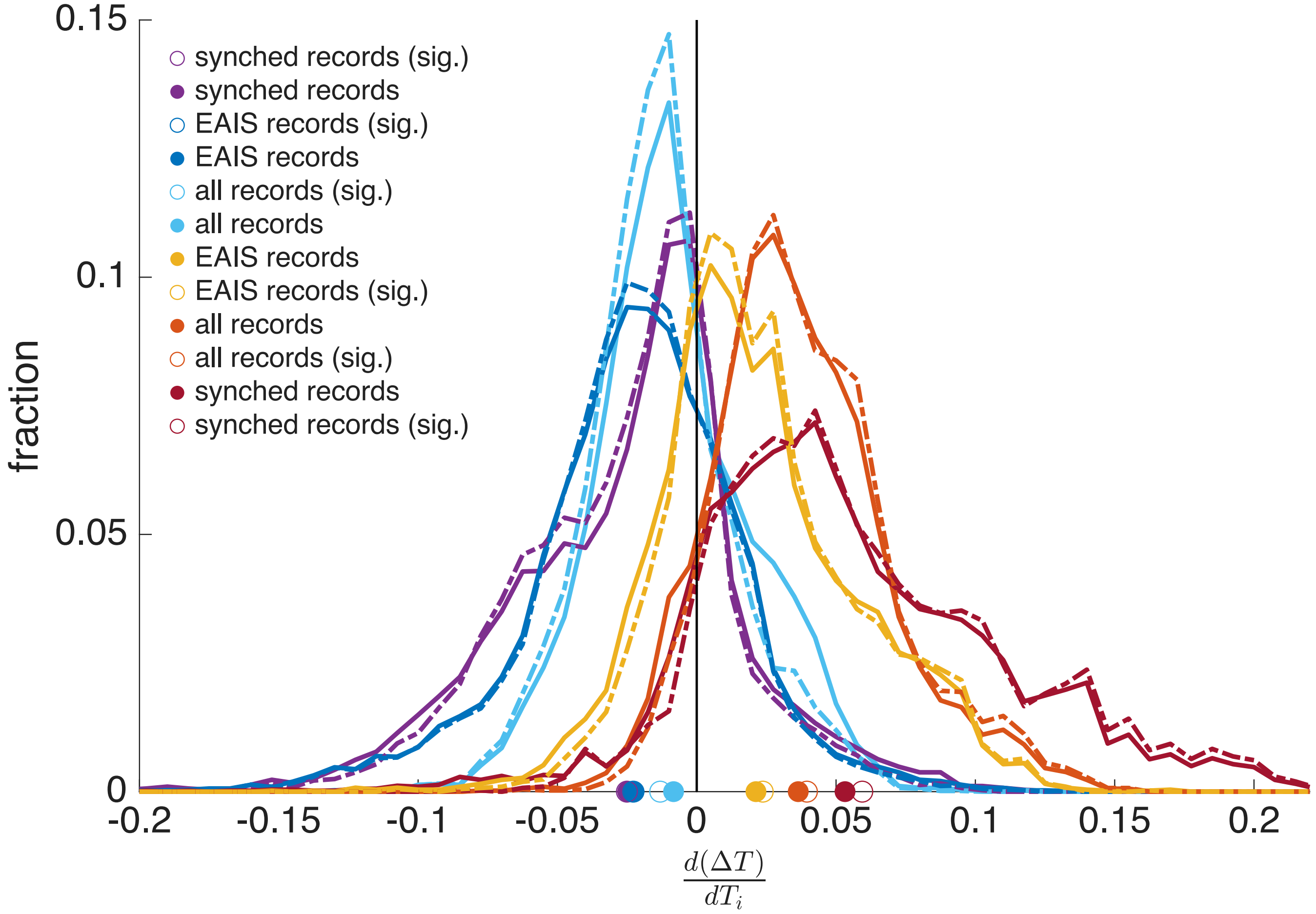

Fig. S11. Histograms of slopes ($\frac{d\Delta T}{dT}$) of the best-fit lines (panel **b** of Figure 4) from the sliding block analysis on the reconstructions, as described in the main text. Slopes are segregated by intervals of continent-mean cooling (blue and purple lines) and warming (yellow and red lines). The mean of each histogram is indicated by colored dots along the x-axis. Six different histograms are plotted for both the warming and cooling intervals. These are derived from examining all records, only those in East Antarctica (labeled "EAIS"), and only those on synchronized age scales (labeled "synched"). For each set of records we plot the histograms for all intervals of change as well as only those where the mean change across all sites ($\overline{\Delta T}$) is statistically distinguishable from zero, using a Student's t-test (dashed lines and open dots along the x-axis).

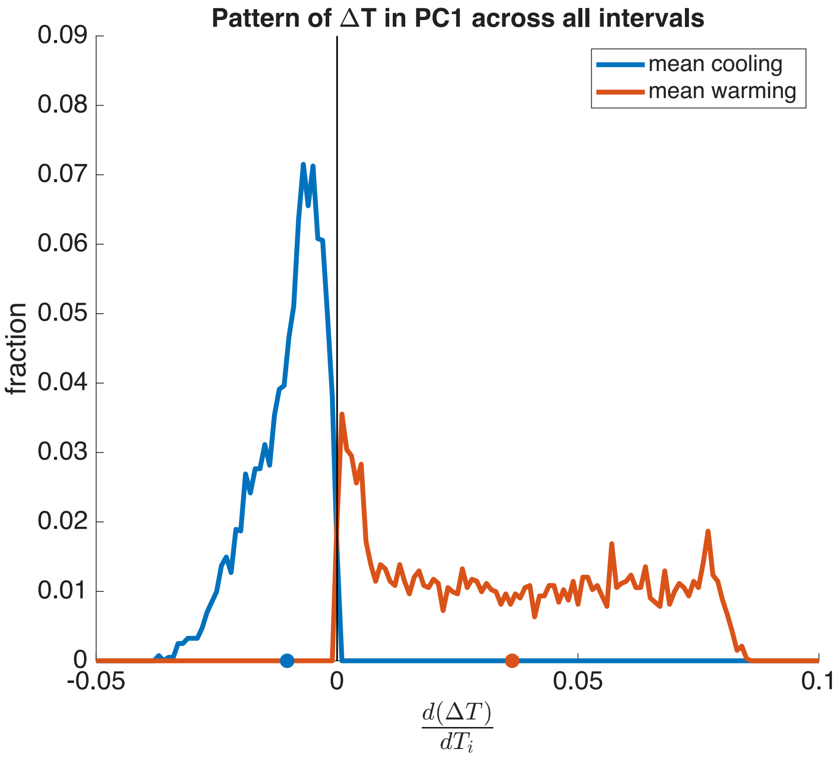

Fig. S12. Histograms of slopes ($\frac{d\Delta T}{dT}$) of the lines of best fit (panel **d** of Figure 4) from the sliding block analysis on PC1. Slopes are segregated by intervals of continent-mean cooling (blue line) and warming (red line). Colored dots along x-axis show mean of each histogram.

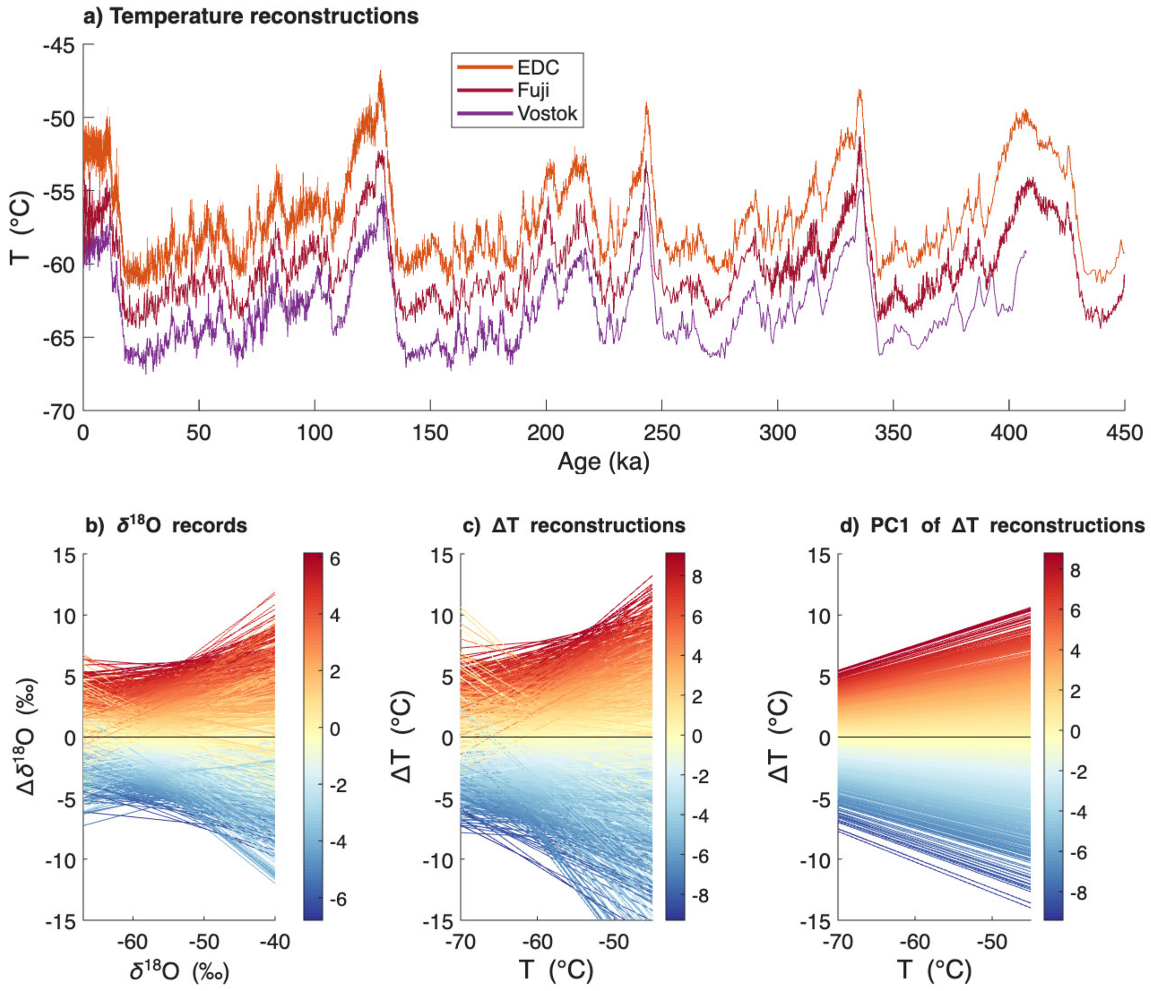

Fig. S13. a) The temperature reconstructions of EDC, Fuji, and Vostok for the last four glacial cycles. Results of the sliding block analysis over the last 400 ka, on **b)** the raw δ^{18}O, **c)** the surface temperature reconstructions, and on **d)** PC1 of the surface temperature reconstructions.

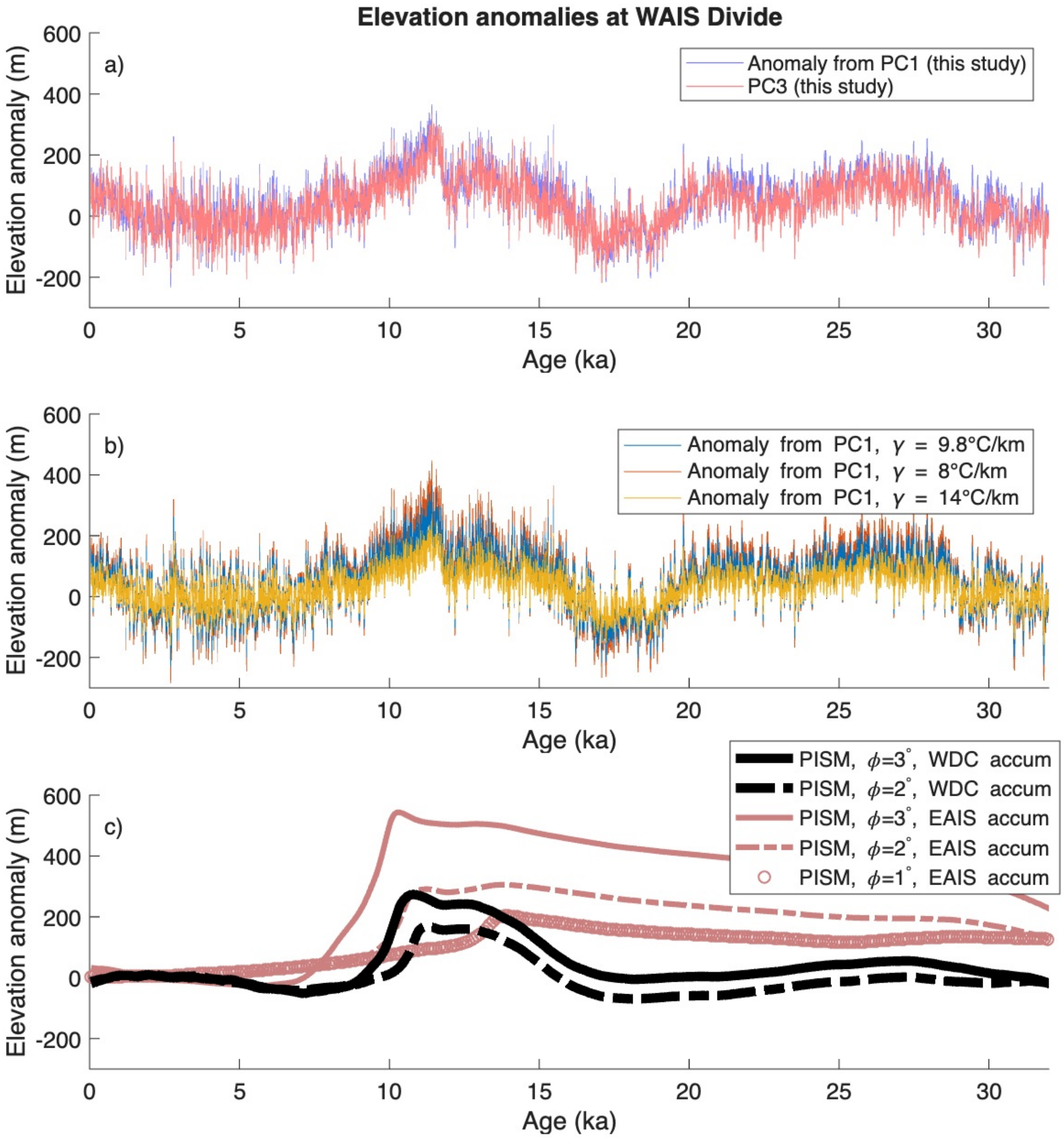

Fig. S14. Sensitivity of elevation changes at WAIS Divide. **a)** Reconstructions of elevation changes at WDC using temperature variability in excess of PC1 (blue) and using PC3 (red), assuming a surface lapse rate of $\gamma = 9.8^{\circ C}/_{km}$. **b)** Reconstructions of elevation changes at WDC using temperature variability in excess of PC1 and the dry adiabatic lapse rate $\gamma = 9.8^{\circ C}/_{km}$ (blue), a sub-adiabatic lapse rate $\gamma = 8^{\circ C}/_{km}$ (red) typical of lower elevations on the continent rather than at WDC, and a super-adiabatic lapse rate $\gamma = 14^{\circ C}/_{km}$ (gold), typical of the high East Antarctic plateau. **c)** PISM simulations from (17, 18) using different critical till angles, ϕ, and accumulation forcing based on East Antarctic records (EAIS) and from the WDC record. All elevation anomalies are in reference to the mean elevation of the most recent 5000 years.

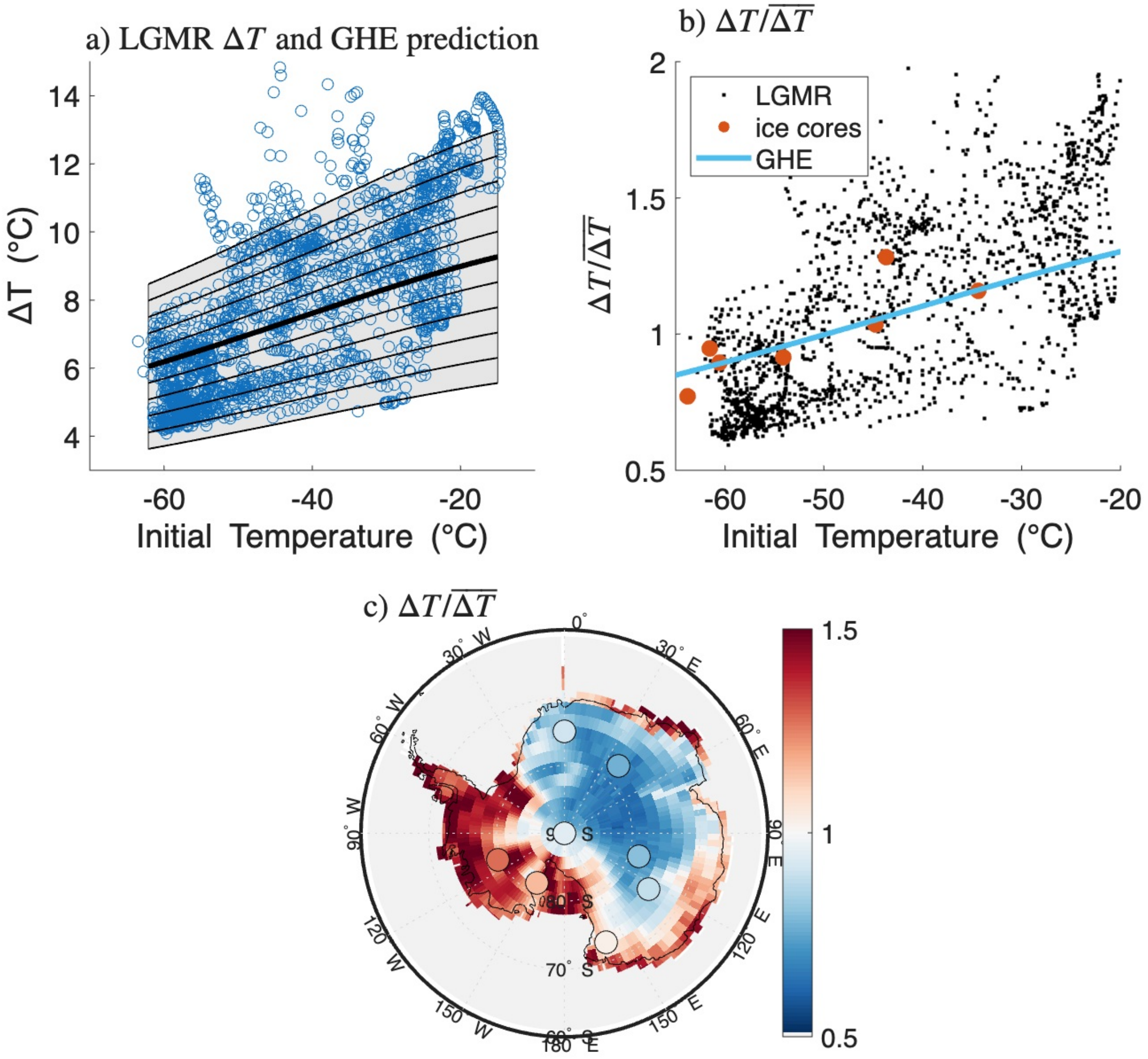

Fig. S15. Comparison to LGMR. **a.** ΔT in the LGMR over Antarctica versus initial surface temperature (blue dots). Black lines show the predicted pattern of warming from the GHE feedback for a mean forcing plus local variability (a spread of ± 5Wm^{-2} in grey). This shows that initial surface temperature is a strong predictor of total warming, as predicted by the GHE feedback, even in the presence of variability in the mean forcing. **b.** Black dots show relative LGM to Holocene temperature change in the LGMR (as in panel a) as a function of LGM surface temperature, red circles show relative temperature change in our ice-core reconstructions, and blue line shows the expected pattern of warming from the GHE feedback given the mean Antarctic temperature change. **c.** Relative LGM to Holocene temperature change in the LGMR as a function of the mean temperature change at all grid points with initial surface temperature between -65℃ and -30℃ (for comparison with ice-core reconstructions). Relative temperature change in our ice-core reconstructions are shown in filled in circles.

References

1. BR Markle, et al., Global atmospheric teleconnections during Dansgaard-Oeschger events. *Nat. Geosci.* **10**, 36–40 (2017).
2. WAIS Divide Project Members, Onset of deglacial warming in West Antarctica driven by local orbital forcing. *Nature* **500**, 440–444 (2013).
3. EJ Steig, et al., Recent climate and ice-sheet changes in West Antarctica compared with the past 2000 years. *Nat. Geosci.* **6**, 372–375 (2013).
4. EJ Brook, et al., Timing of millennial-scale climate change at Siple Dome, West Antarctica, during the last glacial period Siple Dome, West Antarctica, during the last glacial period. *Quat. Sci. Rev.* **24**, 1333–1343 (2005).
5. AS Schilla, "The stable isotopes and deuterium excess from the Siple Dome ice core: implications for the late Quaternary climate and elevation history of the Ross Sea Region, West Antarctica," PhD thesis, University of Colorado at Boulder (2007).
6. B Stenni, et al., The deuterium excess records of EPICA Dome C and Dronning Maud Land ice cores (East Antarctica). *Quat. Sci. Rev.* **29**, 146–159 (2010).
7. A Landais, et al., Interglacial Antarctic–Southern Ocean climate decoupling due to moisture source area shifts. *Nat. Geosci.* **14**, 918–923 (2021).
8. F Vimeux, KM Cuffey, J Jouzel, New insights into Southern Hemisphere temperature changes from Vostok ice cores using deuterium excess correction. *Earth Planet. Sci. Lett.* **203**, 829–843 (2002).
9. R Uemura, et al., Ranges of moisture-source temperature estimated from Antarctic ice cores stable isotope records over glacial-interglacial cycles. *Clim. Past* **8**, 1109–1125 (2012).
10. B Stenni, et al., Expression of the bipolar see-saw in Antarctic climate records during the last deglaciation. *Nat. Geosci.* **4**, 46–49 (2011).
11. EJ Steig, et al., Continuous-flow analysis of δ^{17}O, δ^{18}O, and δD of H_2O on an ice core from the South Pole. *Front. Earth Sci.* **9**, 640292 (2021).
12. BR Markle, EJ Steig, Improving temperature reconstructions from ice-core water-isotope records. *Clim. Past* **18**, 1321–1368 (2022).
13. E Kalnay, et al., The NCEP/NCAR 40-year reanalysis project. *Bull. Am. meteorological Soc.* **77**, 437–471 (1996).
14. MT Chahine, et al., AIRS: Improving weather forecasting and providing new data on greenhouse gases. *Bull. Am. Meteorol. Soc.* **87**, 911–926 (2006).
15. SA Sejas, PC Taylor, M Cai, Unmasking the negative greenhouse effect over the Antarctic Plateau. *NPJ climate atmospheric science* **1**, 17 (2018).
16. AG Pendergrass, A Conley, FM Vitt, Surface and top-of-atmosphere radiative feedback kernels for CESM-CAM5. *Earth Syst. Sci. Data* **10**, 317–324 (2018).
17. T Albrecht, R Winkelmann, A Levermann, Glacial-cycle simulations of the Antarctic Ice Sheet with the Parallel Ice Sheet Model (PISM) – Part 1: Boundary conditions and climatic forcing. *The Cryosphere* **14**, 599–632 (2020).
18. T Albrecht, R Winkelmann, A Levermann, Glacial-cycle simulations of the Antarctic Ice Sheet with the Parallel Ice Sheet Model (PISM) – Part 2: Parameter ensemble analysis. *The Cryosphere* **14**, 633–656 (2020).
